# Pathological oligodendrocyte precursor cells revealed in human schizophrenic brains and trigger schizophrenia-like behaviors and synaptic defects in genetic animal model

**DOI:** 10.1038/s41380-022-01777-3

**Published:** 2022-09-21

**Authors:** Guangdan Yu, Yixun Su, Chen Guo, Chenju Yi, Bin Yu, Hui Chen, Yihui Cui, Xiaorui Wang, Yuxin Wang, Xiaoying Chen, Shouyu Wang, Qi Wang, Xianjun Chen, Xuelian Hu, Feng Mei, Alexei Verkhratsky, Lan Xiao, Jianqin Niu

**Affiliations:** 1 Department of Histology and Embryology, Chongqing Key Laboratory of Neurobiology, Brain and Intelligence Research Key Laboratory of Chongqing Education Commission, Third Military Medical University, Chongqing, China; 2 Research Centre, The Seventh Affiliated Hospital of Sun Yat-sen University, Shenzhen, China; 3 Department of Neurobiology, and Department of Neurology of Sir Run Run Shaw Hospital, Zhejiang University School of Medicine, Hangzhou, China; 4 Department of Neurosurgery, The Second Affiliated Hospital of Third Military Medical University, Chongqing, China; 5 School of Life Sciences, Faculty of Science, University of Technology Sydney, Sydney, Australia; 6 Department of Physiology, College of Basic Medical Science, Chongqing Medical University, Chongqing, China; 7 Faculty of Biology, Medicine and Health, The University of Manchester, Manchester, UK; 8 Achucarro Center for Neuroscience, IKERBASQUE, 48011 Bilbao, Spain

**Keywords:** Schizophrenia, Neuroscience, Cell biology

## Abstract

Although the link of white matter to pathophysiology of schizophrenia is documented, loss of myelin is not detected in patients at the early stages of the disease, suggesting that pathological evolution of schizophrenia may occur before significant myelin loss. Disrupted-in-schizophrenia-1 (DISC1) protein is highly expressed in oligodendrocyte precursor cells (OPCs) and regulates their maturation. Recently, DISC1-Δ3, a major DISC1 variant that lacks exon 3, has been identified in schizophrenia patients, although its pathological significance remains unknown. In this study, we detected in schizophrenia patients a previously unidentified pathological phenotype of OPCs exhibiting excessive branching. We replicated this phenotype by generating a mouse strain expressing DISC1-Δ3 gene in OPCs. We further demonstrated that pathological OPCs, rather than myelin defects, drive the onset of schizophrenic phenotype by hyperactivating OPCs’ Wnt/β-catenin pathway, which consequently upregulates Wnt Inhibitory Factor 1 (Wif1), leading to the aberrant synaptic formation and neuronal activity. Suppressing Wif1 in OPCs rescues synaptic loss and behavioral disorders in DISC1-Δ3 mice. Our findings reveal the pathogenetic role of OPC-specific DISC1-Δ3 variant in the onset of schizophrenia and highlight the therapeutic potential of Wif1 as an alternative target for the treatment of this disease.

## Introduction

Schizophrenia, a complex and severe psychiatric condition [1], is one of the 15 leading causes of disability, affecting 1 in 300 people worldwide [2, 3]. Although highly heritable, the pathophysiological mechanisms of schizophrenia have not been well understood. Clinical evidence has linked white matter abnormalities to schizophrenia symptoms [4]. Pathophysiological changes include impaired oligodendrocyte differentiation, myelination, and white matter loss [5–7]. However, several diffusion tensor imaging (DTI) studies found no changes in the white matter volume during the early stages of schizophrenia [8–11], suggesting that early pathogenesis of schizophrenia may precede significant myelin loss.

DISC1 emerged as a schizophrenia susceptible gene since its genomic disruption, while frameshift mutation, or single nucleotide polymorphisms (SNPs) were associated with familial schizophrenia [12–14]. Further studies revealed that DISC1 acts as a scaffold protein regulating multiple schizophrenia-related neuronal development processes through its protein partners [15–18]. However, recent advances showed that DISC1 dysfunction also affects OPC maturation and subsequent myelination [19, 20], while RNA-sequencing analyses in both humans and mice demonstrated that OPCs express higher levels of DISC1 when compared to neurons or mature oligodendrocytes (OLs) [21, 22], raising a possibility that the high presence of DISC1 in the OPCs may contribute to the pathogenesis of schizophrenia.

In addition, previous studies showed that DISC1 undergoes extensive alternative splicing, which is highly regulated by schizophrenia-associated SNPs [23, 24]. Several DISC1 splicing variants are upregulated in the hippocampi of schizophrenia patients [24–26], where significant pathological changes occur at the clinical high risk (CHR) stage of the onset of schizophrenia [27]. Among these variants, DISC1-Δ3, a splicing variant that lacks exon 3, is one of the most upregulated DISC transcripts in multiple brain regions [24, 26], especially in individuals with intronic SNP rs821597 [24]. DISC1-Δ3 transcript translates into a short DISC1 protein isoform lacking the binding sites with several protein partners [26, 28], yet little is known of its role in oligodendroglia or in the pathogenesis of schizophrenia.

In the present study, we revealed previously unknown pathological hypertrophic OPCs in schizophrenia patients. We were able to replicate these pathological OPCs in a newly generated transgenic mouse strain, which mimics the enhanced DISC1 exon 3 splicing in patients by removing the DISC1 exon 3 from a single allele in oligodendroglial lineage cells. Mechanistically, we demonstrated that mice with enhanced DISC1-Δ3 variant expression in OPCs display schizophrenia-like behaviors and synaptic defects, both driven by the overactivated Wnt/β-catenin-Wnt inhibitory factor 1 (Wif1) cascade. Our results provide an alternative insight into the critical role of malfunctional OPCs in pathogenesis of schizophrenia and highlight a molecular target, Wif1, for developing potential therapeutic strategies.

## Results

### OPCs are hypertrophic in schizophrenia patients

**Fig. 1:**
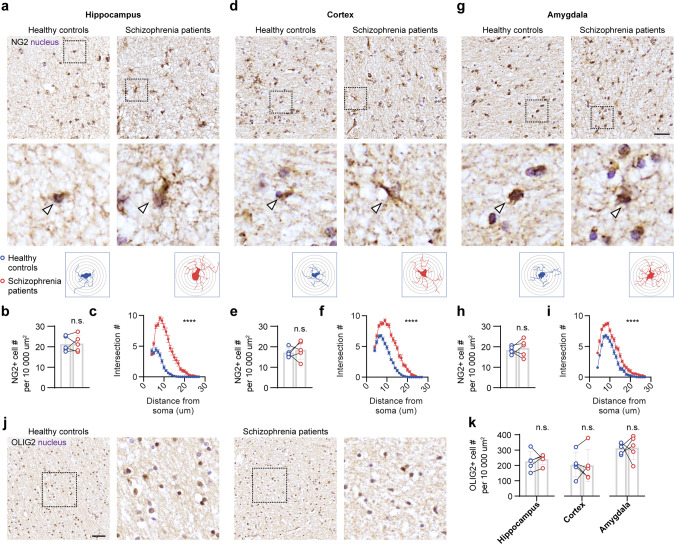
Hypertrophic OPCs in schizophrenia patients. **a** Immunohistochemistry of NG2 in the paraffin-embedded hippocampal sections of healthy controls and schizophrenia patients. Arrowheads highlight NG2^+^ OPCs. Lower panel, example of Sholl analysis. **b** Quantification of NG2^+^ OPC number in the hippocampus. *n* = 5 samples per group. **c** Sholl analysis of NG2^+^ OPC in the hippocampus. *n* = 30 cells from samples per group. **d** Immunohistochemistry of NG2 in the paraffin-embedded cortical sections of healthy controls and schizophrenia patients. Arrowheads highlight NG2^+^ OPCs. Lower panel, example of Sholl analysis. **e** Quantification of NG2^+^ OPC number in the cortex. *n* = 5 samples per group. **f** Sholl analysis of NG2^+^ OPC in the cortex. *n* = 30 cells from samples per group. **g** Immunohistochemistry of NG2 in the paraffin-embedded amygdala sections of healthy controls and schizophrenia patients. Arrowheads highlight NG2^+^ OPCs. Scale bar, 50 µm. Lower panel, example of Sholl analysis. **h** Quantification of NG2^+^ OPC number in the amygdala. *n* = 5 samples per group. **i** Sholl analysis of NG2^+^ OPC in the amygdala. *n* = 30 cells from samples per group. **j** Immunostaining of Olig2 in the hippocampus. Scale bar, 50 µm. **k** Quantification of Olig2 in the hippocampus, cortex, and amygdala. *n* = 5 samples per group. Plots show individual data and mean ± SD, or mean ± SEM in Sholl analysis results. n.s., not significant, *p < 0.05, **p < 0.01, ****p < 0.0001; paired *t*-test, or two-way ANOVA for Sholl analysis.

We examined the histological properties of the OPCs in postmortem brain tissues of schizophrenia patients and age-matched healthy controls (Supplementary Fig. 1a). The Sholl analysis of NG2-positive OPCs revealed previously unidentified hypertrophic morphotype in the hippocampus, prefrontal cortex, and amygdala of schizophrenia patients when compared to the age-matched healthy controls in both human paraffin tissue sections (Fig. 1a-i) and frozen sections (Supplementary Fig. 1b–g). The OPCs in post-mortem tissues of schizophrenia patients displayed a significant increase in the mean number of branches (increased by 73.4 ± 6.9%, 35.3 ± 4.0%, and 21.8 ± 3.9% respectively in the hippocampus, prefrontal cortex, and amygdala in paraffin human brain tissue sections, Fig. 1a–i; increased by 42.1 ± 12.5% and 40.6 ± 14.3%, respectively in the hippocampus and prefrontal cortex in frozen sections, Supplementary Fig. 1b–g) and greater mean branch length (increased by 64.7 ± 7.6%, 31.3 ± 3.1% and 24.9 ± 5.3% in paraffin tissue sections, Fig. 1a–i; increased by 42.9 ± 9.7% and 57.8 ± 13.8% in frozen sections, Supplementary Fig. 1b–g). In contrast, neither the number of NG2-positive OPCs (paraffin section, Fig. 1b, e, h; frozen section, Supplementary Fig. 1c, f) nor the number of OLIG2-positive oligodendroglial cells (paraffin section, Fig. 1j, k) per area unit was affected in schizophrenia patients. This is the first time demonstration of hypertrophic pathological OPCs in schizophrenia patients.

### Enhanced DISC1-Δ3 expression in the oligodendroglia replicates the hypertrophic OPC in patients

DISC1 is a schizophrenia risk gene and regulates oligodendroglial development [19, 20]. The gene sequence of DISC1 is highly conserved between humans and mice (Supplementary Fig. 2a). Re-analyzing RNA-sequencing libraries [21, 22] showed that DISC1 expression in human and mouse OPCs is much higher than in newly-differentiated oligodendrocytes, mature oligodendrocytes, and neurons (Supplementary Fig. 2b). Analysis of mRNA expression of DISC1 splicing variants revealed that Δ3 and Δ7/8 (DISC1 alleles lacking exon 3, or exons 7 and 8) were downregulated during early brain development (Supplementary Fig. 2c), but upregulated in schizophrenia brains [24].

To mimic pathological changes in schizophrenia patients, we generated a DISC1^exon3 flox^ mouse strain, and crossed it with the OPC-specific NG2^CreERT^ mice to obtain NG2^CreERT^:DISC1^exon3 fl/+^ (DISC1-Δ3) mice (Supplementary Fig. 2d). Tamoxifen administration removed a single copy of the DISC1 exon 3 from one allele on the double helix, which replicates enhanced expression of DISC1 exon 3 splicing in schizophrenia patients without affecting the expression of full-length DISC1 gene [23, 24]. The efficiency of genetic modification was confirmed by increased mRNA and protein levels of DISC1-Δ3 in the brain; at the same time expression of full-length DISC1 or DISC1-Δ7/8 remained unchanged (Supplementary Fig. 2e, f). The cell-specific expression of DISC1-Δ3 was confirmed by significantly upregulated mRNA levels selectively in OPCs, but not in other brain cell types (Supplementary Fig. 2g).

**Fig. 2:**
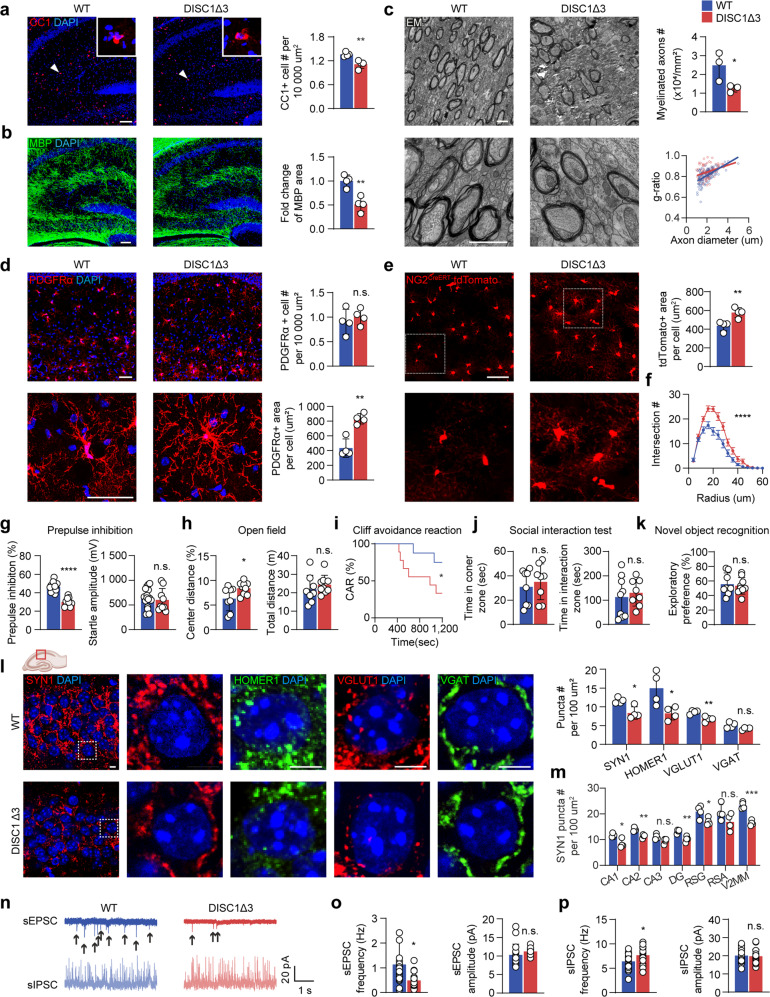
Enhanced DISC1-Δ3 expression in the oligodendroglia phenocopies the hypertrophic OPC in patients and triggers schizophrenia-like behaviors and synaptic defects. WT and DISC1-Δ3 mice were treated with tamoxifen from postnatal day (P)4 to P7 and were analyzed since P23. **a**, **b** Staining and quantification of CC1 and MBP in the hippocampus. Scale bar: 50 µm. *n* = 4 mice. **c** Electron microscopy image of corpus callosum sections and quantification of myelinated axon number and g-ratio. Scale bar, 10 µm. *n* = 3 mice. **d** PDGFRα staining and quantification of PDGFRα^+^ area of each OPC and the number of OPC in the hippocampal CA1 region. Scale bar, 50 µm. *n* = 4 mice. **e** Representative images of NG2^CreERT^:tdTomato, and quantification of the process coverage per cell. Scale bar, 20 µm. *n* = 4 mice. **f** Sholl analysis of NG2^CreERT^:tdTomato in control and DISC1-Δ3 mice. *n* = 30 cells from 4 mice. error bar: SEM. Behavioral tests on WT and DISC1-Δ3 mice, including prepulse inhibition test (**g**), open field test (**h**), cliff avoidance reaction (CAR) test (**i**), social interaction test (**j**), and novel object recognition (**k**). *n* = 8, 9 mice from each group. **l** Staining of synaptic elements, including SYN1 (presynaptic marker), HOMER1 (postsynaptic marker), vGLUT1 (glutamatergic synaptic marker), and vGAT (GABAergic synaptic marker) in the hippocampal CA1 region. Scale bar is 5 µm. Quantification of different synaptic elements in the CA1 region. *n* = 4 mice of each genotype. **m** Quantification of SYN1 in different hippocampal and cortical regions. *n* = 4 mice of each genotype (DG, dentate gyrus. RSG, granular retrosplenial area. RSA, agranular retrosplenial area. V2MM, secondary visual cortex, mediomedial area). **n–p** Representative traces of sEPSCs and sIPSCs recordings from P25 hippocampal slices. Quantification of the frequency and amplitude of sEPSCs. *n* = 15, 18 cells from 3 mice of each genotype. Quantification of the frequency and amplitude of sIPSCs. *n* = 15, 18 cells from 3 mice of each genotype. Plots show individual data and mean ± SD; n.s., not significant, *p < 0.05, **p < 0.01, ***p < 0.001, ****p < 0.0001; two-sided Student’s t-test, or log-rank test for survival curve, two-way ANOVA for Sholl analysis.

Consistent with abnormal myelination detected in familial schizophrenia due to disrupted DISC1 expression [20], the overexpression of DISC1-Δ3 variant in oligodendroglia didn’t affect the total number of oligodendroglial lineage cells and their proliferation (Supplementary Fig. 2i), but impaired oligodendrocyte differentiation (Fig. 2a, b), decreased the number of myelinated axons and reduced the myelin thickness (Fig. 2c). Increased expression of DISC1-Δ3 variant in the newly generated transgenic mouse strain replicated the OPC hypertrophy in schizophrenia patients, as shown by the enlarged PDGFRα (increased by 91.6 ± 16.5%) (Fig. 2d, Supplementary Fig. 2h) and NG2^CreERT^:tdTomato labeling areas per cell (increased by 32.4 ± 8.5%) (Fig. 2e), with a more hypertrophic morphology (Fig. 2f). The morphological appearances of astrocyte, microglia, and vasculature were not affected by increased DISC1-Δ3 variant expression in oligodendroglia (Supplementary Fig. 2j–m).

Taken together, our results reveal hypertrophic morphology of OPCs in both schizophrenia patients and DISC1-Δ3 mice.

### Oligodendroglial DISC1-Δ3 variant triggers schizophrenia-like behaviors and synaptic defects

At postnatal day (P)25 to P36, the age of mice approximately corresponding to human adolescence (12–18 years old) when the first episode of schizophrenia usually occurs [1, 29], enhanced oligodendroglial DISC1-Δ3 splicing resulted in schizophrenia-like sensorimotor gating impairment as reflected by the prepulse inhibition test (Fig. 2g), as well as by the positive symptoms [30] in the open field test and cliff avoidance test (Fig. 2h, i). However, no negative symptoms (assessed by the social interaction test, Fig. 2j), motor deficit (travel distance in the open field test, Fig. 2h), or memory impairments (novel object recognition test, Fig. 2k) were detected in DISC1-Δ3 mice.

Excitation-inhibition disbalance contributes to the pathophysiology of schizophrenia [31, 32]. We found that expression of DISC1-Δ3 in oligodendroglia suppressed synaptogenesis, as indicated by the decreased levels of SYN1^+^ presynaptic and HOMER1^+^ postsynaptic markers in hippocampal and cortical regions at P25 (Fig. 2l, m). Specifically, the number of excitatory synapses labeled with vGLUT1 antibodies but not the inhibitory synapses labeled by vGAT antibodies was reduced in the hippocampal CA1 region (Fig. 2l). These changes recapitulate decreased excitatory/inhibitory synapses ratio observed in schizophrenia patients [31, 32]. In agreement with synaptic alteration, expression of DISC1-Δ3 in cells of oligodendroglial lineage reduced the frequency of spontaneous excitatory postsynaptic currents (sEPSC) and increased the frequency of spontaneous inhibitory postsynaptic currents (sIPSC) (Fig. 2n–p).

To conclude, deletion of DISC1 exon 3 in oligodendroglia is sufficient to induce schizophrenia-like behavioral abnormalities and cause significant synaptic alterations.

### Aberrant OPC but not defective myelin contributes to the onset of schizophrenia-like symptoms in DISC1-Δ3 mice

**Fig. 3:**
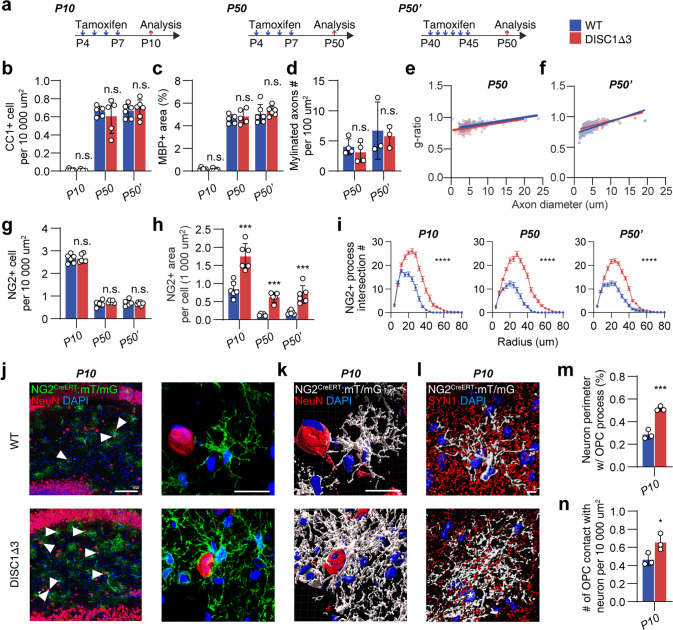
Induction of DISC-Δ3 results in OPCs hypertrophy but does not affect myelination. **a** Three different experiment setups of oligodendroglial DISC-Δ3 induction. **b** Quantification of CC1 numbers in the hippocampus in three different setups. *n* = 5 or 6 mice. **c** Quantification of MBP^+^ area in the hippocampus in three different setups. *n* = 5 or 6 mice. **d** Quantification of myelinated axon numbers in the second and the third setups. *n* = 5 or 6 mice. **e**, **f** g-ratio chart in the second and the third setups. **g**, **h** Quantification of NG2^+^ OPC number and NG2^+^ area per OPC in different setups. *n* = 5 or 6 mice. **i** Sholl analysis of NG2 staining. *n* = 30 cells from 3 mice. Error bar: SEM. **j** Staining of NeuN in NG2^CreERT^: mT/mG brain section. Arrowheads highlight the neuron cell bodies enwrapped by membrane GFP (mGFP)-labeled OPC. Scale bar, 100 µm or 10 µm. **k** 3D reconstruction of high magnification images in **j**. Scale bar, 10 µm. **l** 3D reconstruction of SYN1 staining in the CA1 region of a NG2^CreERT^: mT/mG brain section. Asterisks: the adjacent neuronal nucleus. Scale bar, 5 µm. **m** Quantification of the percentage of neuronal cell body enwrapped by mGFP^+^ OPC processes. *n* = 3 mice. **n** Quantification of the number of neurons with cell body contacted by mGFP^+^ OPC. *n* = 3 mice. Plots show individual data and mean ± SD; n.s. not significant, *p < 0.05, **p < 0.01, ***p < 0.001, ****p < 0.0001; two-sided Student’s *t*-test or two-way ANOVA for Sholl analysis.

Oligodendroglial DISC1-Δ3 affects both OPC and myelin as presented above. To distinguish the contribution of hypertrophic OPCs from the myelin deficiency during the onset of schizophrenia, we investigated conditional DISC1 exon 3 deletion at different ages. First, we induced oligodendroglial DISC1 exon 3 deletion at P4 - P7 and analyzed the neurological outcome at P10, before the occurrence of myelin defects in the hippocampus (Fig. 3a–c, Supplementary Fig. 3a, b). Second, we triggered DISC1 exon 3 deletion between P4 - P7, but analyzed the histological and behavioral changes at P50, when the mature OL numbers, myelin densities and structures in DISC1-Δ3 mice attained a level similar to the wildtype littermates (Fig. 3a–e, Supplementary Fig. 3a–c). However, since we cannot rule out the possibility that myelin defects in other brain regions may affect hippocampal synaptogenesis, or that myelin defects in early life (e.g., the neonatal period in this study) may exert prolonged effects on synaptic development in later life (e.g., puberty in this study), we induced the DISC1-Δ3 deletion at a later stage (between P40 - P45), when the majority of myelin sheaths are established. We assessed the histological and behavioral changes at P50, when no OPC differentiation or myelin deficit was found (Fig. 3a–f, Supplementary Fig. 3a–c). In all three experiments, the NG2^+^ areas per cell in DISC1-Δ3 mice at the endpoints increased by 110.1 ± 20.5%, 359.7 ± 46.7%, and 250.2 ± 51.7% respectively (Fig. 3g, h, Supplementary Fig. 3d, e), with a more hypertrophic morphology as shown by Sholl analysis (Fig. 3i). These OPCs were in close contact with the cell bodies of NeuN^+^ neurons with synaptic defects (Fig. 3j–n). However, the number of Olig2^+^ cells remained unchanged in all three experiments (Supplementary Fig. 3f).

**Fig. 4:**
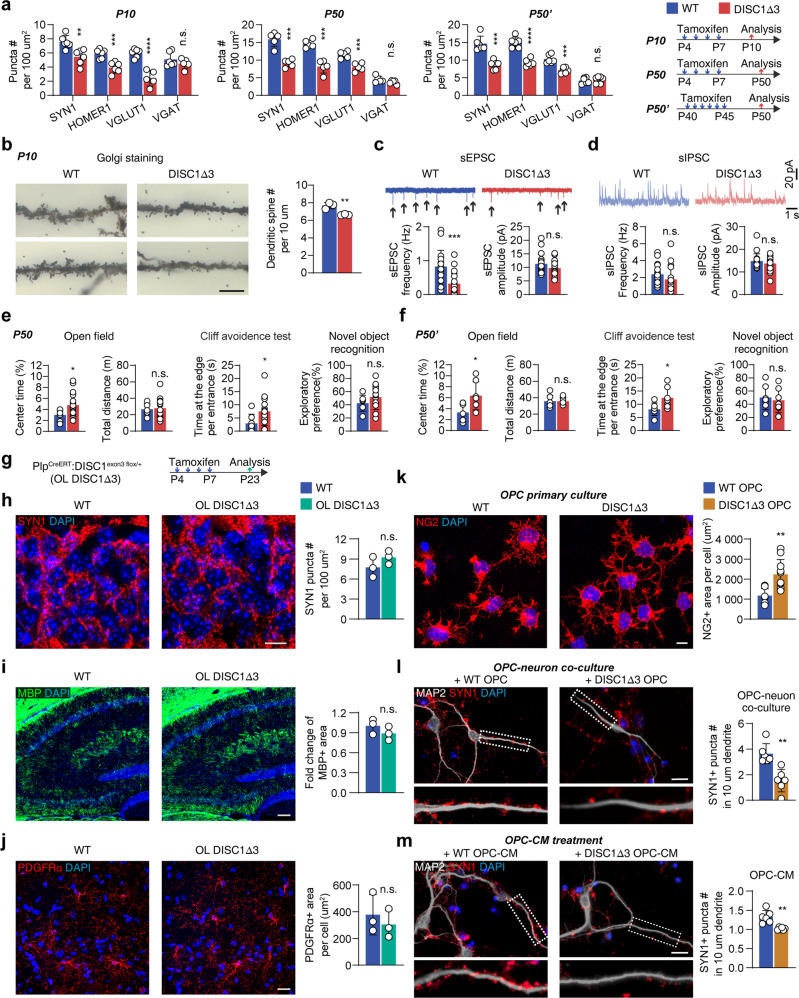
Aberrant OPC but not myelination deficits contribute to the onset of schizophrenia-like symptoms in DISC1-Δ3 mice. **a** Quantification of synaptic elements in CA1 region of three different experiment setups. *n* = 5 or 6 mice. **b** Golgi staining and quantification of the dendritic spines of hippocampal neurons. Scale bar, 5 µm. *n* = 3 mice. **c**, **d** Representative traces of sEPSCs and sIPSCs recording from P10 hippocampal slices and statistics of sEPSCs and sIPSCs. *n* = 19, 20 cells from 3 mice of each genotype. **e**, **f** Behavioral test of mice from the second and the third experiment setups. *n* > 7 mice. **g** PLP^CreERT^ mice were mated to DISC1^exon3 f/+^ mice to produce PLP^CreERT^: DISC1^exon3 f/+^ (OL DISC1-Δ3) mice. Mice were treated with tamoxifen from P4 to P7, and sacrificed at P23 for analysis. **h** Staining and quantification of SYN1 in the hippocampus CA1 region in OL DISC1-Δ3 mice. *n* = 3 mice. **i** Staining and quantification of MBP in OL DISC1-Δ3 mouse hippocampus. *n* = 3 mice. **j** hippocampus. *n* = 3 mice. **k** Staining of NG2 in primary OPC culture and quantification of NG2^+^ area per OPC. Scale bar, 5 µm. *n* = 9 experiments. **l** Staining of MAP2 and SYN1 in primary OPC-neuron co-culture and quantification of SYN1^+^ puncta on dendrite. Scale bar, 10 µm. *n* = 6 experiments. **m** Staining of MAP2 and SYN1 in primary neuron culture treated by OPC-conditioned medium (OPC-CM) and quantification of SYN1^+^ puncta on dendrite. Scale bar, 10 µm. *n* = 6 experiments. Plots show individual data and mean ± SD; n.s. not significant, *p < 0.05, **p < 0.01, ***p < 0.001, ****p < 0.0001; two-sided Student’s *t*-test.

The absence of myelin deficiency allowed us to investigate the contribution of DISC1-Δ3 OPCs to the above mentioned synaptic defects and schizophrenia-like behaviors. We found that pre- and post-synaptic compartments were significantly reduced in the hippocampus in all three experimental groups (Fig. 4a, Supplementary Fig. 4). Golgi staining confirmed decreased number of dendritic spines in DISC1-Δ3 mice at P10 (Fig. 4b). Moreover, enhanced DISC1-Δ3 splicing in OPCs was accompanied with decreased sEPSC frequency as early as at P10 (Fig. 4c, d), while deletion at both early and later ages caused behavioral changes at P50 (Fig. 4e, f). These results indicate that interfering with OPC DISC1-Δ3 expression alone is sufficient to induce synaptic dysfunction and schizophrenia-like behaviors.

In order to further confirm that aberrant OPCs but not mature OLs contribute to the neuronal defects during the onset of schizophrenia, we cross-bred the DISC1^exon3 flox^ mice with the OL-specific PLP^CreERT^ mice to obtain PLP^CreERT^:DISC1^exon3 fl/+^ mice (OL DISC1-Δ3, Fig. 4g). Tamoxifen administration specifically removed a single copy of the DISC1 exon 3 from one allele in OLs (DISC1-Δ3 OLs, Fig. 4g). In the hippocampus of these mice at P21, enhanced expression of DISC1-Δ3 in OLs did not change the numbers of SYN1^+^ synaptic puncta (Fig. 4h). In addition, neither the morphology of PDGFRα^+^ OPCs, MBP^+^ myelin structures, nor numbers of CC1^+^ mature OLs and Olig2^+^ oligodendroglial lineage cells (Fig. 4i, j; Supplementary Fig. 3g, h) were affected. These results suggest an OPC-specific manner to initiate schizophrenia-like pathological changes in the DISC1-Δ3 mice.

Subsequently, we performed in vitro experiments to confirm the direct impact of DISC1-Δ3 OPCs on neurons. Primary OPCs were isolated from the brains of DISC1-Δ3 and non-CreERT wild-type mice at P7 by immunopanning. They were either co-cultured with hippocampal neurons, or used for medium conditioning to treat the hippocampal neurons. Cultured DISC1-Δ3 OPCs showed hypertrophic morphology similar to that observed in DISC1-Δ3 mice (Fig. 4k). Neuronal synapse formation in cells co-cultured with DISC1-Δ3 OPC or exposed to DISC1-Δ3 OPC generated conditioned medium was significantly suppressed (the number of synapses decreased by 58.0 ± 13.3% and 23.5 ± 5.2% respectively, Fig. 4l, m).

Collectively, our results demonstrate that pathological OPCs and not myelin defects are the main contributors to the onset of schizophrenia-like symptoms. In addition, DISC1-Δ3 OPCs affect neuronal synaptic formation in a paracrine manner.

### Hyperactivity of Wnt/β-catenin pathway in OPCs from schizophrenia patients and DISC1-Δ3 mice

**Fig. 5:**
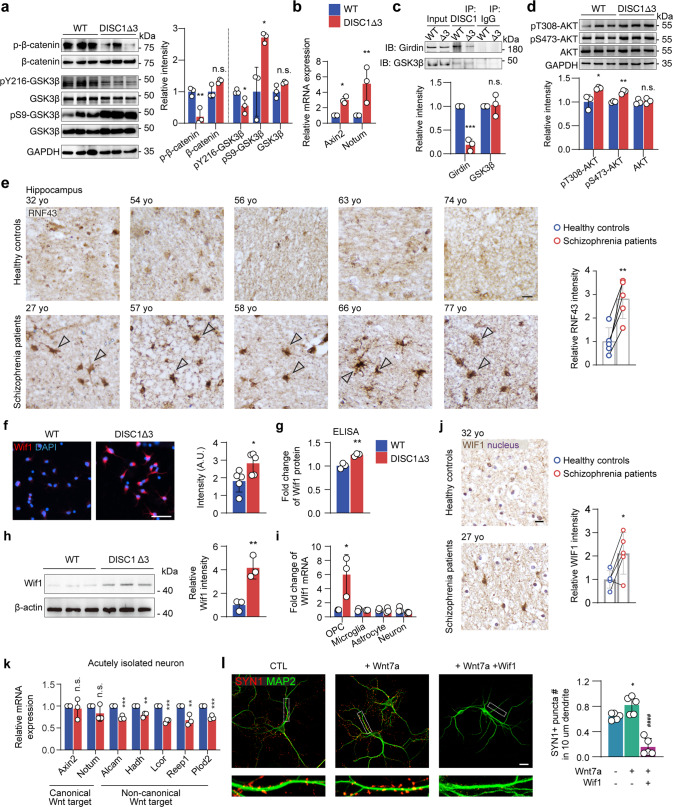
Aberrant Wnt/β-catenin activation in DISC1-Δ3 OPCs upregulates Wif1 expression and suppresses synaptogenesis. **a** Western blot and quantification of β-catenin and GSK3β phosphorylation in isolated WT and DISC1-Δ3 OPCs. *n* = 3 mice. **b** RT-qPCR of canonical Wnt pathway targets Axin2 and Notum in isolated OPCs. *n* = 3 mice. **c** Co-immunoprecipitation experiments in primary WT or DISC1-Δ3 OPCs using anti-DISC1 or normal IgG antibodies, followed by immunoblotting with Girdin and GSK3β antibody. Lower panel is the quantification of Girdin and GSK3β band intensity in DISC1 pull-down normalized to Input. *n* = 3 experiments. **d** Western blot and quantification of AKT phosphorylation in isolated WT and DISC1-Δ3 OPCs. *n* = 3 mice. **e** Representative images of RNF43 staining in each control and schizophrenia patient hippocampal sections. Arrowheads highlight RNF43^+^ cells. Scale bar, 50 µm. Quantification of RNF43 intensity. *n* = 5 age-matched samples, paired t-test for age-match human samples. **f** Staining and quantification of Wif1 in cultured OPCs. Scale bar, 50 µm. *n* = 5 experiments. **g** ELISA of Wif1 in WT or DISC-Δ3 mouse brain lysates. *n* = 3 mice. **h** Western blot and quantification of Wif1 expression in OPCs. *n* = 3 mice. **i** RT-qPCR of Wif1 expression in acutely isolated OPC, microglia, astrocytes, and neurons from WT or DISC-Δ3 mice. *n* = 3 mice. **j** Immunohistochemistry and quantification of Wif1 in hippocampal slice of controls and schizophrenia patients. Arrowheads highlight WIF1^+^ cells. *n* = 5 samples per group. **k** RT-qPCR experiment of acutely isolated neurons from WT and DISC1-Δ3 mice to detect expression of canonical and non-canonical Wnt pathway target gene expression. *n* = 3 mice. **l** Staining of SYN1 and MAP2 in primary hippocampal neurons cultured with or without Wnt7a or Wif1. Right: quantification of SYN1 puncta on dendrites. CTL, control. Scale bar, 10 µm. *n* = 5 experiments. Plots show individual data and mean ± SD; n.s., not significant, *p < 0.05, **p < 0.01, ***p < 0.001; #, statistical analysis between Wnt7a treatment and Wnt7a + Wif1 treatment. ^####^*p* < 0.0001; two-sided Student’s *t*-test.

To gain an insight into underlying molecular mechanisms, RNA-sequencing was performed in the OPCs from DISC1-Δ3 and non-CreERT wildtype mice (Supplementary Fig. 5a–c). The Kyoto Encyclopedia of Genes and Genomes (KEGG) analysis indicated significantly increased activity of Wnt/β-catenin pathway in DISC1-Δ3 OPCs (Supplementary Fig. 5c). This was deduced from a 56.9 ± 14.0% reduction in phosphorylated β-catenin (the degraded form of Wnt signaling stimulator), 44.8 ± 15.0% reduction in autophosphorylation of glycogen synthase kinase 3 β (GSK3β) at Tyrosine 216 (Y216, the functional form of an inhibitor of the Wnt/β-catenin pathway), and a 76.3 ± 20.3% increase in the phosphorylated GSK3β at Serine 9 (S9, Fig. 5a) that inactivates GSK3β [33–35]. In addition, a significant upregulation of Wnt downstream targets Axin2 and Notum was detected (199.3 ± 25.7% and 412.2 ± 133.7% increase, respectively, Fig. 5b), which also indicates an overactivated Wnt/β-catenin pathway in DISC1-Δ3 OPCs.

Subsequently, we investigated how DISC1-Δ3 deletion affects Wnt/β-catenin signaling in the OPCs. DISC1 is known to directly interact with GSK3β and suppress its Y216 autophosphorylation [16, 35]. Thus, DISC1 can inhibit GSK3β function and consequently activate Wnt/β-catenin pathway [16]. Furthermore, DISC1 can also bind to the C-terminal (amino acids 347–854) of Girdin to prevent activation of AKT [36], a suppressor of GSK3β by phosphorylating its S9 residue [36, 37], resulting in the indirect regulation of Wnt/β-catenin pathway. Co-immunoprecipitation analysis showed that DISC1-Δ3, which contains one of the GSK3β binding sites (amino acids 1–220), was still able to directly interact with GSK3β to suppress GSK3β Y216 autophosphorylation (Fig. 5c). However, DISC1-Δ3 structurally lacks the binding site for Girdin and thus cannot bind to it (Fig. 5c), which enables Girdin to activate AKT signaling [36]. Consistently, an increased activity of AKT in DISC1-Δ3 OPCs was also observed (Fig. 5d), which explains the elevated GSK3β S9-phosphorylation. These findings indicate that DISC1-Δ3 is unable to bind to Girdin and therefore, it inhibits GSK3β activity indirectly through the Girdin-AKT pathway, resulting in increased Wnt/β-catenin signaling (Supplementary Fig. 5d).

We further confirmed the hyperactivation of Wnt/β-catenin pathway in the OPCs, from the hippocampus of schizophrenia patients, by detecting an increased immunoreactivity of Wnt downstream gene RNF43 (181.4 ± 30.5% increase, Fig. 5e). This is consistent with a recent finding that upregulation of RNF43 specifically in OPCs correlates with hyperactivated Wnt-signaling in injuries [38, 39]. In this regard, we postulate that the DISC1-Δ3 in OPCs mediates a ‘gain-of-function’ to promote the hyperactivity of the Wnt/β-catenin signaling pathway in schizophrenia brains.

However, the morphological changes in OPCs cannot be achieved either in vivo or in vitro by OPC-specific conditional deletion of the obligate Wnt pathway inhibitor, adenomatous polyposis coli (APC) (Supplementary Fig. 5e, f). This suggests that Wnt/β-catenin pathway is probably not involved in generation of the DISC1-Δ3 OPCs morphological phenotype.

### Wnt-driven Wif1 overexpression impairs synaptic formation

As Wnt/β-catenin pathway activity governs the expression of downstream genes in multiple cellular processes [40], we investigated how hyperactive Wnt signaling in DISC1-Δ3 OPCs affects synaptic formation. Differential expression analysis showed that DISC1-Δ3 altered the expression of several secretory protein-coding genes (Supplementary Fig. 5g, h), especially Wif1 (420.1 ± 21.4-fold increase, Supplementary Fig. 5i), which is a downstream target of Wnt and an inhibitor of the Wnt pathway [41]. Recently, it has been proposed that upregulated Wif1 forms part of a negative feedback mechanism to counteract the excessive activities of the Wnt signaling pathway in OPCs [42].

Increased expression of Wif1 was detected by immunostaining, ELISA, western blot, and qPCR (55.2 ± 21.4%, 23.3 ± 3.9%, 100.2 ± 38.9%, and 500.0 ± 163.1% increase, respectively, Fig. 5f-i). Consistent with our findings in DISC1-Δ3 mice, WIF1 intensity was significantly increased by 111.4 ± 34.5% in the hippocampus of schizophrenia patients when compared to that in the age-matched healthy controls (Fig. 5j), which is in agreement with previous observations that WIF1 is altered in schizophrenia postmortem brains [43, 44].

Since Wif1 is a secreted factor, the overproduction of Wif1 in OPCs may affect other Wnt-dependent processes in adjacent neurons, such as synaptogenesis [45]. To address this question, we measured the activation of Wnt signaling pathway in acutely isolated neurons from P10 DISC1-Δ3 and non-CreERT wildtype mice. We found that the target genes of the non-canonical Wnt pathways [46] were downregulated in the neurons from DISC1-Δ3 mice, whereas the canonical Wnt/β-catenin pathway remains unchanged (Fig. 5k). Next, we treated primary wild-type hippocampal neurons with the synaptogenic Wnt ligand Wnt7a [47] in the presence of Wif1 protein. Wif1 treatment resulted in an 80.8 ± 10.6% reduction in the number of SYN1^+^ synaptic puncta compared to Wnt7a-treatment alone (Fig. 5l). This was accompanied by a decrease in Wnt/Ca^2+^ signaling elements: 55.9 ± 5.0% reduction in CaMK II phosphorylation and suppressed mRNA expression of non-canonical Wnt target genes (Supplementary Fig. 5j, k). Similar changes occur in neurons from DISC1-Δ3 mice, which agrees with previous studies that the canonical and non-canonical Wnt pathways regulate synaptogenesis [48–50]. Therefore, our data suggest that the overproduction of Wif1 protein from DISC1-Δ3 OPCs results in the inhibition of synaptogenesis mediated by non-canonical Wnt pathway.

### Downregulation of Wif1 rescues synaptic defects and behavioral abnormalities in DISC1-Δ3 mice

**Fig. 6:**
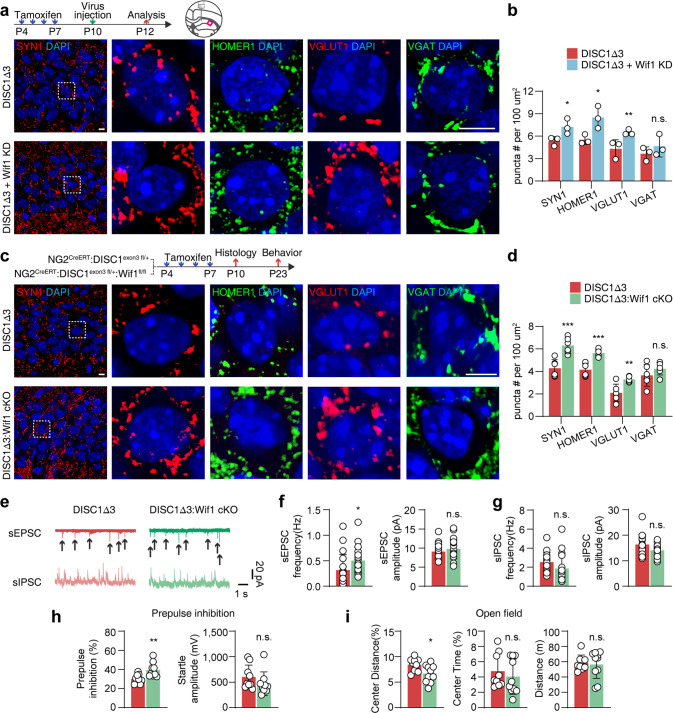
Suppressing expression of oligodendroglial Wif1 rescues synaptic loss and behavioral disorders in DISC1-Δ3 mice. **a** Staining of synaptic elements in the hippocampus of P12 DISC1-Δ3 mice with Wif1-shRNA encoded retrovirus injection. Scramble shRNA encoded retrovirus serves as control. Scale bar, 5 µm. **b** Quantification of synaptic elements in **a**. *n* = 3 mice from each genotype. **c** Staining of synaptic elements in the hippocampus of P10 Wif1 conditional KO DISC1-Δ3 Wif1 conditional KO mice. Scale bar, 5 µm. **d** Quantification of synaptic elements in **c**. *n* = 6 mice from each genotype. **e–g** Representative results of sEPSC or sIPSC recording in P10 Wif1 conditional KO DISC1-Δ3 mice. Quantification of sEPSC frequency and amplitude. *n* = 18, 19 cells from 3 mice of each genotype. Quantification of sIPSC frequency and amplitude. *n* = 18, 19 cells from 3 mice of each genotype. **h** Prepulse inhibition test of P23 Wif1 KO DISC1-Δ3 mice. *n* = 9, 10 mice of each genotype. **i** Open field test of P23 Wif1 conditional KO DISC1-Δ3 mice. *n* = 9, 10 mice of each genotype. Plots show individual data and mean ± SD; n.s. not significant, *p < 0.05, **p < 0.01, ***p < 0.001; two-sided Student’s *t*-test.

To further verify whether manipulating Wif1 expression can rescue synaptic defects and behavioral abnormalities in DISC1-Δ3 mice, we knocked down Wif1 in the DISC1-Δ3 OPCs by microinjecting Wif1 shRNA retrovirus into the hippocampus [51]. The infection efficiency was confirmed by the co-expression of retrovirus-driven GFP and the NG2^creERT^-driven tdTomato (Supplementary Fig. 6a). The infection specificity for oligodendroglial cells was validated by co-labeling of GFP^+^ with NG2^creERT^:tdTomato^+^ (82.13 ± 1.45%) and PDGFRα^+^ (45.19 ± 1.11%), but rarely with the markers of neurons (5.03 ± 0.38%), microglial cells (5.44 ± 0.67%), astrocytes (0.69 ± 0.69%), and pericytes (1.33 ± 0.67%) (Supplementary Fig. 6a), which is consistent with previous observations [51]. Western blot and ELISA showed that Wif1 knockdown decreased Wif1 protein levels in the hippocampus (Supplementary Fig. 6b, c), and improved synaptic formation by 35.4 ± 12.7%, as judged by the SYN1^+^ puncta quantification (Fig. 6a, b). However, Wif1 knockdown did not change the total number of oligodendroglial lineage cells, oligodendroglial differentiation, degree of myelination, the number of neurons or OPC morphology (Supplementary Fig. 6d–h).

Subsequently, we conditionally knocked out Wif1 in the OPCs of DISC1-Δ3 mice (Supplementary Fig. 6i). This did not change the number, differentiation, or hypertrophic morphology of OPCs, the number of neurons (Supplementary Fig. 6j–n). However, such conditional knockout increased synaptogenesis as evidenced by a 47.1 ± 9.8% increase in SYN1^+^ puncta (Fig. 6c, d). In particular, we observed an increase in the number of vGLUT1 labeled excitatory synapses while the number of vGAT-positive inhibitory synapses remained unchanged (Fig. 6c, d). Furthermore, Wif1 knockout reversed neuronal activity alterations, as demonstrated by restored sEPSC in hippocampal neurons (Fig. 6e–g). Wif1 conditional knockout also mitigated schizophrenia-like behavioral abnormalities such as increased prepulse inhibition and decreased center distances in the open field test in DISC1-Δ3 mice, (Fig. 6h, i), suggesting an improvement in behavioral abnormalities [52].

Taken together, our results from the DISC1-Δ3 mouse model indicate that Wnt-hyperactivation in the OPCs can disrupt synapse formation and initiate the pathogenesis of schizophrenia by overproduction of Wnt inhibitor Wif1. As a result, the downregulation of Wif1 in DISC1-Δ3 OPCs restored synaptic formation and improved neuronal function.

## Discussion

We identified hypertrophic OPCs as a new histopathological signature of schizophrenic brains in both human patients and mouse model, provided compelling evidence that expression of DISC1 exon 3 splicing variant solely in OPCs is sufficient to suppress synaptogenesis, and presented a potential mechanism by which the onset of schizophrenia related to OPCs could be driven by the overproduction of Wif1, a Wnt-pathway inhibitor, in response to the hyperactivation of Wnt/β-catenin signaling in DISC1-Δ3 OPCs.

Evidence gathered previously suggests that myelin defects contribute to pathophysiology of schizophrenia [7]. However, several DTI studies on young patients showed no changes in white matter at the onset of disease [8–11]. This contradicts the current hypothesis postulating that schizophrenic phenotypes originate from myelin defects, and hints at an alternative possibility that myelin abnormalities may not be necessarily responsible for the onset of disease in young schizophrenia patients, but develop as the disease progresses [8–11]. Our in-vivo and in-vitro findings demonstrate that abnormal OPC function initiates neuronal malfunction. In particular, expression of a single gene variant DISC1-Δ3 in the OPCs is sufficient to trigger the onset of schizophrenia-related pathological changes, which may explain the results of previous imaging studies. Schizophrenia is usually diagnosed between late adolescence and early thirties [53, 54], when myelination is still ongoing [55, 56]. Any abnormal activation of Wnt/β-catenin pathway in OPCs within this age may affect their differentiation and hinder myelination [57, 58]. Indeed, our findings in schizophrenia patients and mouse model show that Wnt-signaling elements in the OPCs are hyperactive especially at the initiation of schizophrenia-like pathology in mice, suggesting that defects in myelination may follow later when disease evolves [9, 10]. Although we still lack direct evidence linking OPC hypertrophies to Wnt-signaling hyperactivation, as well as their association with DISC1-Δ3 splicing in schizophrenia patients, these early changes in OPCs, (found in both schizophrenia patients and mouse model), may either initiate or contribute to the onset of schizophrenia.

Our study further corroborates the multi-functional role of OPCs in the central nervous system (CNS). In other neurological disorders, such as multiple sclerosis, hypoxic-ischemic encephalopathy, and psychiatric diseases, OPCs not only contribute to aberrant myelin formation [39, 57], but are also involved in the regulation of blood-brain barrier integrity [42], CNS immune regulation [59, 60] and the behavioral outcomes [61, 62]. In the present study, we provided new evidence that selective enhancement of DISC1 exon 3 splicing in OPCs, disrupts the formation of vGLUT1^+^ excitatory synapse but not vGAT^+^ inhibitory synapse. This leads to excitatory/inhibitory synaptic imbalance (E/I imbalance), which significantly contributes to the pathophysiology of schizophrenia [63]. The E/I imbalance is well documented in the hippocampus of schizophrenia patients, where the excitatory synaptic elements are significantly reduced, while the inhibitory synaptic elements are less affected [64–66]. Hence, our study proposes an alternative non-cell-autonomous mechanism where OPC malfunction suppresses excitatory synaptogenesis thus contributing to pathogenesis of schizophrenia. Both excitatory and inhibitory synapses also connect neurons to OPCs [67, 68], it is tempting to examine whether the neuron-OPC synapses are also disrupted in schizophrenia in future studies.

Mechanistically, we found that DISC1-Δ3 significantly enhances the activation of Wnt/β-catenin pathway by regulating GSK3β, the Wnt/β-catenin pathway inhibitor, both directly and indirectly. The DISC1-Δ3 protein retains GSK3β binding region, and therefore can directly interact with GSK3β to suppress its Y216 residue autophosphorylation and catalytic activity [16, 35], although the underlying mechanism requires further scrutiny. On the other hand, it has been reported that GSK3β S9 can be phosphorylated by several protein kinases, such as AKT (PKB) [37, 69]. While AKT activity can be suppressed by DISC1 due to its competitive binding to Girdin, the AKT co-activator [36], we found that the AKT pathway is activated by DISC-Δ3 variant lacking the Girdin binding region, thus resulting in an indirect enhancement of GSK3β S9 phosphorylation. By analyzing both direct and indirect regulation, we provided new insights into how DISC1-Δ3 stabilizes β-catenin and promotes Wnt/β-catenin signaling in the OPCs, which may act as a starting point of schizophrenia pathogenesis. This new mechanism is in agreement with observations showing altered Wnt pathway genes such as GSK3β, WIF1, as well as AKT1 in schizophrenia postmortem brains [43, 44, 70]. Notably, manipulating Wif1 production alone improves synaptogenesis and mitigates schizophrenia-like behaviors in DISC-Δ3 mice. Consequently, DISC-Δ3 represents a perspective target for novel treatment strategies. DISC1 can also regulate neurogenesis, neurite outgrowth, and synaptic plasticity, which are all involved in the pathogenesis of schizophrenia [16, 36, 71, 72]. These processes however could also be regulated by many other factors, and hence the relative contribution of DISC1 requires further studies.

Notably, OPCs hypertrophy is not linked to the overactivation of Wnt/β-catenin pathway in DISC1-Δ3 mice, as our experiments, as well as previous studies, failed to induce such morphological change by increasing Wnt/β-catenin signaling pathway activity. Changes in OPCs morphology may be caused by DISC1 acting as a scaffolding protein, with the DISC1-Δ3 variant changing the cytoskeletal dynamics through AKT/mTOR signaling [36, 73]. Such possibility was supported by our KEGG pathway analysis. Alternatively, prolonged inhibition of GSK3β can cause cell hypertrophy [74]. Be it as it may, morphological changes correlate with the functional changes in OPCs, and thus OPCs hypertrophy may be used as a histological mark for early-stage schizophrenia.

In conclusion, our findings provide new evidence of the pathological potential of OPCs in the onset of schizophrenia in genetically susceptible individuals, whereas targeting Wif1 in OPCs may open a new direction in developing effective therapeutic strategies for schizophrenia.

## Methods

### Human schizophrenia tissues and immunohistochemical staining

Human schizophrenia and healthy comparable post-mortem tissue slides were provided by the National Health and Disease Human Brain Tissue Resource Center at Zhejiang University in China (S2019017). All human tissues were collected following fully informed consent by the donors via a prospective donor scheme following ethical approval by the Human Ethics committee of Zhejiang University School of Medicine (#2020–005). Cases assessed are described in Supplementary Fig. 1a. The immunohistochemical technique has been described previously [42], with some modifications. Images were captured by using VS200 Research Slide Scanner (Olympus) or Axio Imager M2 with the apotome system (Zeiss).

### Mice and behavioral tests

R26-LSL-tdTomato mice were crossed with NG2^CreERT^ mice [75] to visualize cell morphology. The DISC1 exon3-flox mice were crossed with NG2^CreERT^ mice or PLP^CreERT^ mice [76] to generate NG2^CreERT^: DISC1^exon3 fl/+^ (DISC1-Δ3) or PLP^CreERT^: DISC1^exon3 fl/+^ (DISC1-Δ3 OL) conditional knockout (cKO) mice. Wif1-flox mice were crossed with DISC1-Δ3 mice to generate NG2^CreERT^: DISC1^exon3 fl/+^: Wif1^fl/fl^ cKO mice. PDGFRα^creER^ mice [77] or Olig2^cre^ mice [78] were crossed with APC^fl/fl^ mice [79] to over-activate the Wnt pathway in OPCs. The behavioral changes of DISC1-Δ3 mice were evaluated by Prepulse inhibition test, Open field test, Cliff avoidance reaction test, Social interaction test, and Novel object recognition Test.

### Hippocampus brain slice preparation and electrophysiological recordings

Brains transverse slices (300 μm) were cut on a vibratome (Leica VT1200S) and stored in a recording solution at room temperature. For hippocampus neuron spontaneous excitatory postsynaptic currents (sEPSCs) and inhibitory postsynaptic currents (sIPSCs) recording, whole-cell patch-clamp recordings were made with borosilicate glass pipettes. Cells were visualized with infrared optics on an upright microscope (BX51WI, Olympus). A MultiClamp 700B amplifier and pCLAMP10 software were used for electrophysiology (Axon Instruments).

### Wif1 shRNA Retrovirus and Stereotaxic surgery

To silence *Wif1* expression in vivo, three shRNA sequences targeting different sites of *Wif1* mRNA were designed, and a scrambled shRNA target sequence was designed as a negative control. The shRNA sequences were inserted into the previously described retrovirus vector [51]. Using stereotaxic techniques, Wif1 shRNA Retrovirus was injected bilaterally into the hippocampus CA1 of the P10 mouse. Two days post retrovirus injection, mice were perfused with 4% buffered paraformaldehyde.

### Quantification methods

Images captured with the VS200 Research Slide Scanner and FV3000 confocal microscope were used to quantify cell numbers. To quantify positive cell numbers, cell counting was conducted on nine randomly chosen fields for each sample using an Image Pro Plus image analysis system. The number of cells in a fixed area in the CA1 region was quantified manually. To quantify the fluorescence positive area per cell, a threshold was set to include the fluorescence positive signal, followed by quantification of the area of positive signals in the region of interest; the number of positive cells in the region of interest was counted, and the positive area per cell was calculated as: positive area/the number of cells. The Sholl analysis was performed using a Fiji Sholl Analysis plugin described previously [80]. This measurement was only done for OPCs in which the cell processes were clearly seen. Quantification of synapse puncta was done using images captured by the FV3000 confocal microscope, which was then analyzed using the Fiji software. The fluorescent intensity and Western-blot positive band, the positive areas were automatically selected in image-Pro Plus 5 software. The areas of interest (AOI) were separated by setting the threshold at least two times the background.

### Statistical analysis

Statistical significance between groups was determined using the GraphPad Prism software 9.0 (GraphPad Software, San Diego, CA, USA). Data were presented as means ± standard deviation (SD). Fold changes reported in the text are (mean difference ± SEM of difference) % compared to the controls. The unpaired *t*-test was used to determine the significance between two experimental groups, while one-way analysis of variance (ANOVA) was used to determine statistical significance when comparing multiple groups. All statistical tests were two-tailed. *p*-values less than 0.05 were considered statistically significant. Significant statistical results are indicated as: *p < 0.05, **p < 0.01, ***p < 0.001, ****p < 0.0001. No statistical methods were used to pre-determine sample sizes but our sample sizes are similar to those reported in the previous publications [42]. No sample or animal was excluded from the analysis. Transgenic mouse litters were randomly subjected to different experiment paradigm. The investigators were blinded when analyzing the data. Data distribution was assumed to be normal, but this was not formally tested. The F-test was conducted to compare the variance between the groups during statistical analysis. All experiments were performed at least 3 times, and the findings were replicated in individual mice and cell cultures in each experiment.

## Supplementary information

Supplementary information

## Data Availability

All data are available in the main text or the supplementary materials. RNA-seq data have been deposited in the NCBI GEO under the accession number GSE183341.
