# Supplementary material for: Pathological oligodendrocyte precursor cells revealed in human schizophrenic brains and trigger schizophrenia-like behaviors and synaptic defects in genetic animal model
Source: Mol Psychiatry. 2022 Sep 21;27(12):5154–66. doi: 10.1038/s41380-022-01777-3 (PMC9763102; doi:10.1038/s41380-022-01777-3)
Supplement: Supplementary file 1 — Supplementary information [file 41380_2022_1777_MOESM1_ESM.doc]

**Pathological oligodendrocyte precursor cells revealed in human schizophrenic brains and trigger schizophrenia-like behaviors and synaptic defects in genetic animal model**

Guangdan Yu^1, 9^, Yixun Su^1, 2, 9^, Chen Guo^4^, Chenju Yi^2^, Bin Yu^1, 3^, Hui Chen^5^, Yihui Cui^4^, Xiaorui Wang^1^, Yuxin Wang^1^, Xiaoying Chen^1^, Shouyu Wang^1^, Qi Wang^1, 2^, Xianjun Chen^6^, Xuelian Hu^1^, Feng Mei^1^, Alexei Verkhratsky^7,8^ *, Lan Xiao^1, 3^ *, Jianqin Niu^1, 10^ *

^1^ Department of Histology and Embryology, Chongqing Key Laboratory of Neurobiology, Brain and Intelligence Research Key Laboratory of Chongqing Education Commission, Third Military Medical University, Chongqing, China.

^2^ Research Centre, The Seventh Affiliated Hospital of Sun Yat-sen University, Shenzhen, China.

^3^ Department of Neurosurgery, The Second Affiliated Hospital of Third Military Medical University, Chongqing, China.

^4^ Department of Neurobiology, and Department of Neurology of Sir Run Run Shaw Hospital, Zhejiang University School of Medicine, Hangzhou, China.

^5^ School of Life Sciences, Faculty of Science, University of Technology Sydney, Sydney, Australia.

^6^ Department of Physiology, College of Basic Medical Science, Chongqing Medical University, Chongqing, China.

^7^ Faculty of Biology, Medicine and Health, The University of Manchester, Manchester, UK.

^8^ Achucarro Center for Neuroscience, IKERBASQUE, 48011 Bilbao, Spain

^9^ These authors contributed equally.

^10^ Lead Contact.

* Corresponding author Email: [Alexej.Verkhratsky@manchester.ac.uk](mailto:Alexej.Verkhratsky@manchester.ac.uk) (A.V.); [xiaolan35@tmmu.edu.cn](mailto:xiaolan35@tmmu.edu.cn ) (L. X.); [jianqinniu@tmmu.edu.cn](javascript:void(0)) (J. N.).

**Supplementary methods**

**Human schizophrenia tissues and immunohistochemical staining**

Human schizophrenia and healthy comparable post-mortem tissue slides were provided by the National Health and Disease Human Brain Tissue Resource Center at Zhejiang University in China (S2019017). All human tissues were collected following fully informed consent by the donors via a prospective donor scheme following ethical approval by the Human Ethics committee of Zhejiang University School of Medicine (#2020-005). Cases assessed are described in Figure S1A.

The immunohistochemical technique has been described previously; with some modifications^1^. Briefly, the human paraffin tissue sections were deparaffinized by immersion in fresh xylene twice for 10 min each, rehydrated with a grade descending series of ethanol concentrations, and treated with 3% H_2_O_2_ for 10 min at RT to eliminate the endogenous peroxidase. Antigen retrieval was performed in citrate buffer (pH 6.0) and microwaved at high power for 2 min. After blocking with 5% bovine serum albumin and 0.2% Triton X-100 for 1 hour at room temperature, the samples were incubated with primary antibodies: rabbit anti-NG2 (Millipore, AB5320), rabbit anti-RNF43 (Abcam, ab217787), rabbit anti-OLIG2 (Millipore, AB9610), rabbit anti-WIF1 (Abcam, ab186845) overnight at 4℃. Next, biotinylated goat anti-rabbit IgG (BOSTER, SA2002, China) was applied at 37°C for 30 min, and an additional incubation of slides in streptavidin- peroxidase complex (BOSTER, SA2002, China) was performed for 30 min at 37°C. Then, 3,3′-diaminobenzidine (Abcam, ab64238) was used for chromogenic detection. The frozen sections of human tissues were blocked with 5% bovine serum albumin and 0.2% Triton X-100 for 1 hour at room temperature, followed by being labeled with primary antibody rabbit anti-NG2 (Millipore, AB5320) overnight at 4℃. The immunoreaction was visualized by DAB. Images were captured by using VS200 Research Slide Scanner (Olympus) or Axio Imager M2 with the apotome system (Zeiss).

**Mice**

All mice were maintained on a 12 h/12 h light/dark cycle with free access to food and water. All animal procedures were conducted under institutional guidelines and protocols approved by the animal welfare and ethics committee of the third military medical university at Chongqing. The C57BL/6 wild-type mice were purchased from the university animal breeding center.

NG2^CreERT^ mice have been described previously^2^. R26-LSL-tdTomato mice (The Jackson Laboratory, 007908) or mT/mG mice (The Jackson Laboratory, 007676) were crossed with the NG2^CreERT^ mice to validate the oligodendroglial-specific targeting and to visualize cell morphology.

The DISC1 exon3-flox and Wif1-flox mice were generated de novo using the CRISPR-Cas9 system to insert loxP sites franking DISC1 exon3 and Wif1, respectively, in C57BL/6 mouse zygotes, which were then transferred to pseudo-pregnant CD1 mice at the blastocyst stage. These procedures were performed by Gempharmatech. Co., Ltd, China. The mice were then crossed with NG2^CreERT^ mice to generate NG2^CreERT^: DISC1^exon3 fl/+^ (DISC1-Δ3) or NG2^CreERT^: DISC1^exon3 fl/+^: Wif1^fl/fl^ conditional knockout (cKO) mice; or crossed with PLP^CreERT^ mice^3^ to generate PLP^CreERT^: DISC1^exon3 fl/+^ (DISC1-Δ3 OL) cKO mice.

PDGFRα^creER^ mice^4^ or Olig2^cre^ mice^5^ were crossed with APC^fl/fl^ mice^6^ to over-activate the Wnt pathway in OPCs.

To induce Cre-mediated recombination, the mice were given tamoxifen (10 mg/kg, Sigma-Aldrich, T5648) force-fed for 4 consecutive days (P4-P7) or 6 consecutive days (P40-P45).

**Behavioral Tests**

Prepulse inhibition test: the acoustic startle response was measured with automated startle chambers (MED ASSOCIATES, INC, MED-ASR-PRO1) as previously described^7^. Briefly, each test began with a habituation phase (10 min with a constant 65 dB background noise), Startle responses to a 120 dB auditory stimulus were measured for a period of 40 ms. The test session consisted of 45 trials with no stimulus (background white noise only), 120 dB pulse of 40 ms duration, or 120 dB pulse preceded 40 ms by a 75 dB pulses of 20 ms in duration. Trials were given in random order with variable intervals (20–60 s) between each trial. Percentage of prepulse inhibition (%PPI) was calculated as %PPI = 100 × [(pulse alone score) − (prepulse + pulse score)] ∕ pulse alone score.

Open field test: The open field test was performed to determine locomotor activity and anxiety-like behavior, mice were placed in the center of an open field box (50 × 50 × 50 cm) (Biowill, Shanghai, China), and their activity was recorded for 20 min. The time and distances traveled in the center zone, as well as the total distances traveled, were measured.

Cliff avoidance reaction test: This test was performed to evaluate the impulsive behavior as described previously^8^. A round platform (diameter of 16 cm; thickness of 2 cm) was set up at the height of 50 cm, and each mouse was then gently placed on the platform and the duration (20 min) that it remained on the platform was recorded.

Social interaction test: The test was performed as described previously^9^. Briefly, the test was divided into two phases, each of which lasted 10 min. In the ﬁrst phase, the test mice were placed in the middle of the box and allowed to move freely in the chamber for 10 min while two empty wire cages were placed in the left and right of the chamber. In the second phase, a C57BL/6J stranger mouse that had never been exposed to the test mice was placed in one wire cage, the test mouse was allowed to freely explore the chamber for 10 min. To determine whether the mouse was susceptible or resilient, the time spent in the interaction zone and the time spent in the no interaction zone were recorded.

Novel object recognition Test: The experimental apparatus was an open-field chamber (25 cm × 25 cm × 40 cm). Mice were individually habituated in the empty chamber for 2 days (5 min per day) before testing. During the test, two identical objects were placed into the chamber and the mice were allowed to explore freely for 5 min. Two hours later, one of the objects was replaced by a novel object, the mouse was re-introduced into the apparatus and recorded 5min, a ratio of the amount of time spent in exploring the novel object over the total time spent in exploring both objects was measured.

**Hippocampus brain slice preparation and electrophysiological recordings**

The method has been described previously^10^. Animals were rendered unconscious by 4% isoflurane in the air. Brains were removed and placed for 30s in an ice-cold and oxygenated artificial ACSF containing 125 mM NaCl, 2.5 mM KCl, 25 mM NaHCO_3_, 1.25 mM NaH_2_PO_4_, 1 mM MgCl_2_, 1 mM CaCl_2_, 25 mM glucose, 1 mM sodium pyruvate and continuously gassed with 95% O_2_ and 5% CO_2_. Transverse slices (300 μm) were cut on a vibratome (Leica VT1200S), incubated in ACSF at 32℃ with oxygenated (95% O_2_ and 5% CO_2_) at least 30 min and stored in a recording solution at room temperature. For hippocampus neuron spontaneous excitatory postsynaptic currents (sEPSCs) and inhibitory postsynaptic currents (sIPSCs) recording, whole-cell patch-clamp recordings were made with borosilicate glass pipettes (PC-100, Narishige; 5-6 MΩ) in voltage-clamp mode and at the holding potential (HP) of –70 mV (sEPSC) and 0mV (sIPSC). Recording pipettes were filled with internal solution of the following composition (mM): 125 mM Cs-methane sulphonate, 8 mM NaCl, 2 mM Mg-ATP, 0.3 mM Na_3_-GTP, 0.3 mM EGTA, 10 mM HEPES and 10 mM Na-phosphocreatine (pH = 7.25). Cells were visualized with infrared optics on an upright microscope (BX51WI, Olympus). A MultiClamp 700B amplifier and pCLAMP10 software were used for electrophysiology (Axon Instruments).

**Primary cell cultures**

OPCs and other brain cell types were isolated from cerebral hemispheres of P7 mouse pups by immunopanning as previously described^11^. Immunopanning antibodies used were: OPC, Pdgfrα antibody (Abcam, ab96569); microglia, CD45 antibody (BD Pharmingen™, ab550539); astrocyte, Integrin beta 5 antibody (eBioscience, 14-0497-82); neuron, p75 NGF receptor antibody (Abcam, ab52987).

These purified mouse OPCs were cultured in poly-D-lysine-coated 10 cm dishes or 24-well plates with coverslips for experiments. In the OPC culture medium, PDGF-AA (10ng/ml, Peprotech, 100-13A) was used to stimulate OPC proliferation and was removed from the culture medium to induce OPC differentiation. The cells on coverslips were fixed for immunostaining, proteins were collected for Western blot. The purified mouse OPCs were cultured for 4 days for cultured medium collection. The collected medium was filtered with a 0.22 µm filter (Millipore), then collected as the conditioned medium for downstream experiments or stored at -80℃.

For primary neuronal culture, mouse hippocampal neurons were isolated from E15-17 mouse embryos. Cells were resuspended in Neurobasal medium with B27, GlutaMax, and 1 mM HEPES, supplemented with 10% FBS, filtered through a 70 µm cell strainer, and plated onto poly-L-lysine coated coverslips. The medium was replaced with neuron culture medium the next day, and AraC (10 µM) was added to the cell culture to kill proliferating cells on day 2, and then replaced with neuron culture medium again on day 3.

For OPC-neuron co-culture, the purified OPCs were seeded onto primary neurons in 24-well plates on day 9 and maintained in OPC-neuron culture medium (50% neuron culture medium and 50% OPC medium without PDGF-AA).

For neuron treatments, Wnt7a (100ng/ml, Peprotech, 120-31), Wif1 (1ug/ml, Abcam, ab208465), or Wnt7a with Wif1 were used to incubate with primary cultured neurons in 24-well plates on day 9 for 24 hours. PBS treatment was used as the control. To test the effects of OPC-conditioned medium on neurons, primary cultured neurons were treated with conditioned medium (25% fresh medium+ 75% conditioned medium) for 24 hours.

**Fluorescence-activated cell sorting (FACS)**

Isolation of pericytes from mouse brain was conducted using FACS as described previously with minor modification ^12^. Briefly, isolated mouse brains were minced and digested by collagenase and dispase. Tissue was then triturated, and the resulting suspension was loaded onto 22% Percoll, followed by centrifugation to remove myelin and other debris. Cells recovered from the pellet were subjected to immunolabelling with the following antibodies: PerCP/Cyanine5.5 anti-mouse CD45 (Biolegend, 103132), FITC anti-mouse CD31(Biolegend, 102405), APC anti-mouse CD140b (Biolegend, 136008). Immunolabeled cells were then subjected to FACS to obtain CD140b+ CD31- CD45- pericytes.

**Immunohistochemistry**

The method has been described previously^1^. Primary antibodies used were: Rabbit anti-NeuN (Abcam, ab177487), Rabbit anti-c-Fos (CST, 2250S), Rabbit anti-BLBP (Abcam, ab32423), Rabbit anti-NG2 (Millipore, MAB5320), Rat anti-PDGFRα (BD Biosciences, 558774), Rabbit anti-Olig2 (Millipore, AB9610), Mouse anti-CC1 (Millipore, OP80), Rat anti-MBP (Millipore, MAB386), Rabbit anti-Iba1 (Wako, 019-19741), Rabbit anti-Ki67 (Thermo, MA514520), Goat anti-GFP (Abcam, ab5450), Rat anti-PDGFRβ (Invitrogen, 14-1402-81), Rabbit anti-Synapsin1 (CST, #5297), Rabbit anti-Homer1(Synaptic Systems,#160003), Mouse anti-Vgat (Millipore,#AB5062P), Guinea pig anti-VGLUT1 (Millipore, #AB5905), Chicken anti-MAP2 (Millipore, AB5543), Lectin (Vector Laboratories, Inc. DL-1174), Mouse anti-Fibrinogen (Abcam, ab58207). Images were captured using VS200 Research Slide Scanner (Olympus), FV3000 confocal microscope (Olympus), SpinSR confocal microscope (Olympus), or Axio Imager M2 with the apotome system (Zeiss).

**RNA-seq**

RNA-seq experiments were performed on freshly isolated OPCs from the DISC1-Δ3 and WT mice at P7. OPCs were isolated by immunopanning as described previously^11^. RNA was extracted from the isolated cells by Trizol (Thermo) according to the manufacturer’s protocol. RNA-seq was performed by the Beijing Genomics Institute (BGI). Differential expression analysis was performed using DESeq2.

**ELISA**

To examine the concentrations of Wif1 proteins in brain lysates, mouse Wif1 Elisa Kit (CUSABIO, CSB-EL026113MO) was used according to the manufacturer’s instructions. The OD values were determined by measuring the absorbance at 450 nm using a microplate reader (Bio-RAD, Model 680). Independent experiments were performed in triplicates.

**RT-qPCR**

Total RNA was isolated by TRIZOL® combined with the RNeasy Plus Mini Kit (Qiagen, 74134) according to the manufacturer’s protocol. cDNA was synthesized from 1 μg of total RNA. Quantitative polymerase chain reaction (qPCR) was performed with the Real-time PCR Detection System (Roche) and FastStart Universal SYBR® Green Master (Roche, 04913850001). RT-qPCR primer sequences for DISC1 variant expression analyses were listed as follows: DISC1 full (forward 5′-GAGGATGGCGATTACGATACTG-3′, reverse 5′-AGAGCAGGTTGCTGTGAAG-3′, span from exon3 to exon4); DISC1-Δ3 (forward 5′-GAGCAGAGACATTGAGACAGAG-3′, reverse 5′-CTGCCAGGTAACCCAAGAA-3′, span from exon2 to exon 2-exon4 boundary); DISC1-Δ7/8 (forward 5′-CAGGAGGAAGCTGCTTCTCC-3′, reverse 5′-CAGGAACATCTCTAGCCCTTC-3′, span from exon5 to exon6-exon9 boundary).

**Western blot**

To determine the protein expression pattern of DISC1, Wif1, p-GSK-3β, GSK-3β, p-β-catenin, and β-catenin in OPCs and brain tissue, western blot analysis was performed. Briefly, 40-80 μg of protein were separated by 10% SDS-PAGE and then electrophoretically transferred to nitrocellulose membrane. Membranes were probed by antibodies including rabbit anti-DISC1 (Abcam, ab192258), rabbit anti-Wif1 (Abcam, ab155101), mouse anti-p-GSK-3β (Ser9) (CST, #14630), rabbit anti-GSK-3β (CST, #9315), rabbit anti- p-β-catenin (Ser33/37/Thr41) (CST, #9561), rabbit anti-β-catenin (CST, #8480), rabbit anti-p-SAPK/JNK (Thr183/Tyr185) (CST, #4668), rabbit anti-SAPK/JNK (CST, #9252), rabbit anti-p-CaMKII(Thr286) (CST, #12716), rabbit anti-CaMKll alpha (Abcam, ab92332) and mouse anti-β-actin (Beyotime, AF0003). Protein bands were visualized by chemiluminescence (ECL Plus, GE Healthcare) after incubation with HRP-conjugated secondary antibodies. Images were captured by ChampChemi 610 Chemiluminescence Imaging System (Beijing Sage Creation, China). Protein band intensity was analyzed using the Image Pro Plus software.

**Wif1 shRNA Retrovirus and Stereotaxic surgery**

To silence Wif1 expression in vivo, three [shRNA](https://www.sciencedirect.com/topics/neuroscience/small-hairpin-rna) sequences targeting different sites of Wif1 mRNA were designed, and a scrambled shRNA target sequence was designed as a negative control. The target sequences were: shRNA1, CCAACTGTCAATGTCCCTT; shRNA2, GCTCAACCACCTGCTTTAA; shRNA3, GGGATCCACCTGAATCCAA. The shRNA sequences were inserted into the previously described retrovirus vector^13^. Using stereotaxic techniques, pROV-U6-shRNA1(Wif1)-EF1A(S)-EGFP-3Flag [pROV-U6-shRNA (Scramble)-EF1A(S)-EGFP-3Flag as negative control] was injected bilaterally into the hippocampus CA1 of the P10 mouse at the following coordinates: 1.5 mm posterior to bregma, 0.8 mm from the midline and 1.8mm ventral to the surface of the skull. The total volume injected was 1 μL (1.5e5 TU). The needle was left in place for an additional 10 min after injection. Two days post retrovirus injection, mice were perfused with 4% buffered paraformaldehyde.

**References**

1. Niu J, Tsai HH, Hoi KK, Huang N, Yu G, Kim K *et al.* Aberrant oligodendroglial-vascular interactions disrupt the blood-brain barrier, triggering CNS inflammation. *Nature neuroscience* 2019; **22**(5)**:** 709-718.

2. Zhu X, Hill RA, Dietrich D, Komitova M, Suzuki R, Nishiyama A. Age-dependent fate and lineage restriction of single NG2 cells. *Development (Cambridge, England)* 2011; **138**(4)**:** 745-753.

3. Doerflinger NH, Macklin WB, Popko B. Inducible site-specific recombination in myelinating cells. *Genesis (New York, NY : 2000)* 2003; **35**(1)**:** 63-72.

4. Kang SH, Fukaya M, Yang JK, Rothstein JD, Bergles DE. NG2+ CNS glial progenitors remain committed to the oligodendrocyte lineage in postnatal life and following neurodegeneration. *Neuron* 2010; **68**(4)**:** 668-681.

5. Schüller U, Heine VM, Mao J, Kho AT, Dillon AK, Han YG *et al.* Acquisition of granule neuron precursor identity is a critical determinant of progenitor cell competence to form Shh-induced medulloblastoma. *Cancer cell* 2008; **14**(2)**:** 123-134.

6. Robanus-Maandag EC, Koelink PJ, Breukel C, Salvatori DC, Jagmohan-Changur SC, Bosch CA *et al.* A new conditional Apc-mutant mouse model for colorectal cancer. *Carcinogenesis* 2010; **31**(5)**:** 946-952.

7. Willott JF, Carlson S, Chen H. Prepulse inhibition of the startle response in mice: relationship to hearing loss and auditory system plasticity. *Behav Neurosci* 1994; **108**(4)**:** 703-713.

8. Yamashita M, Sakakibara Y, Hall FS, Numachi Y, Yoshida S, Kobayashi H *et al.* Impaired cliff avoidance reaction in dopamine transporter knockout mice. *Psychopharmacology (Berl)* 2013; **227**(4)**:** 741-749.

9. Han QQ, Yang L, Huang HJ, Wang YL, Yu R, Wang J *et al.* Differential GR Expression and Translocation in the Hippocampus Mediates Susceptibility vs. Resilience to Chronic Social Defeat Stress. *Frontiers in neuroscience* 2017; **11:** 287.

10. Yang Y, Cui Y, Sang K, Dong Y, Ni Z, Ma S *et al.* Ketamine blocks bursting in the lateral habenula to rapidly relieve depression. *Nature* 2018; **554**(7692)**:** 317-322.

11. Zhang Y, Chen K, Sloan SA, Bennett ML, Scholze AR, O'Keeffe S *et al.* An RNA-sequencing transcriptome and splicing database of glia, neurons, and vascular cells of the cerebral cortex. *J Neurosci* 2014; **34**(36)**:** 11929-11947.

12. Crouch EE, Doetsch F. FACS isolation of endothelial cells and pericytes from mouse brain microregions. *Nat Protoc* 2018; **13**(4)**:** 738-751.

13. Chen TJ, Kula B, Nagy B, Barzan R, Gall A, Ehrlich I *et al.* In Vivo Regulation of Oligodendrocyte Precursor Cell Proliferation and Differentiation by the AMPA-Receptor Subunit GluA2. *Cell Rep* 2018; **25**(4)**:** 852-861 e857.

**Supplementary figures**


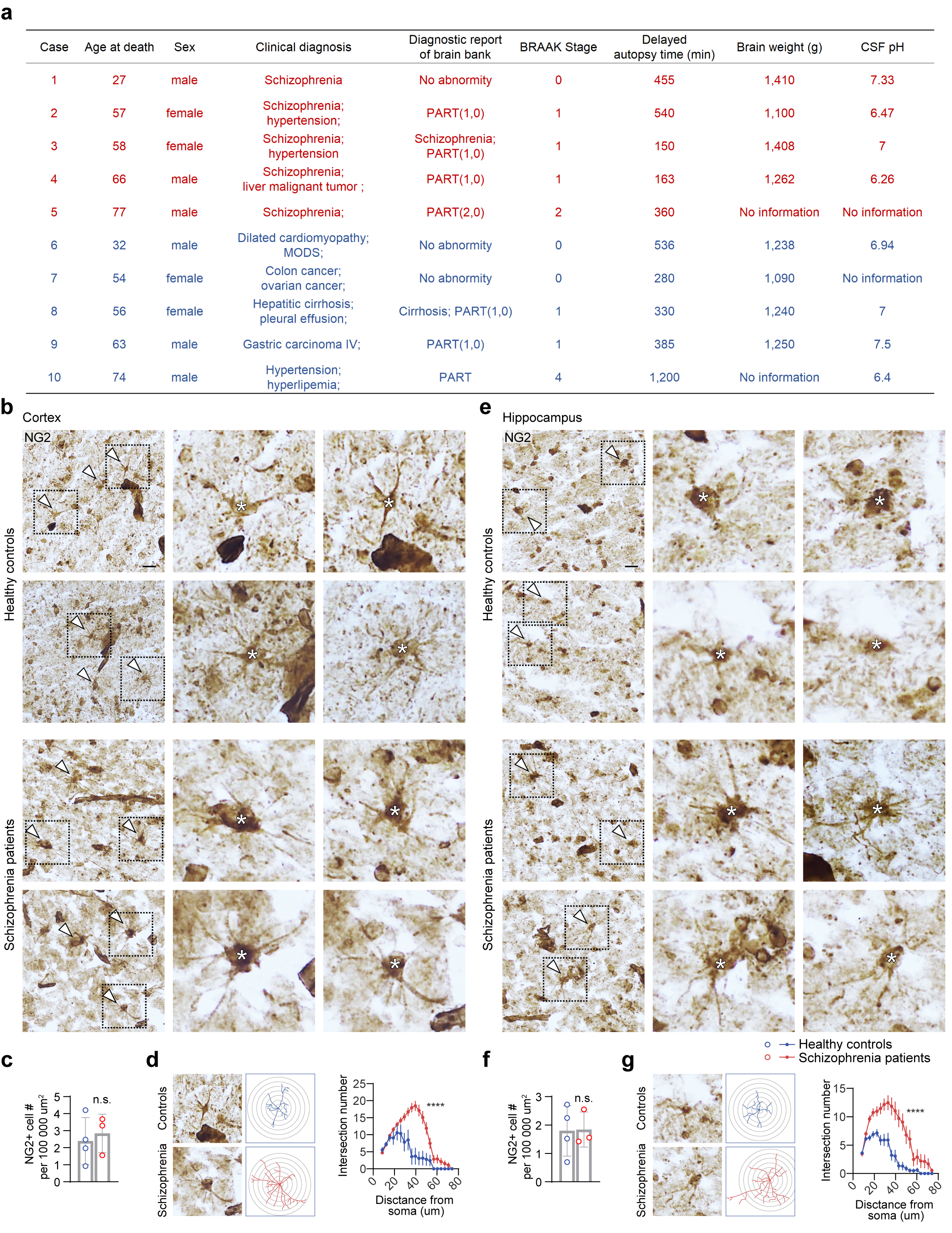


**Supplementary figure 1. Hypertrophic OPCs in schizophrenia patients. a** Information of human samples. Schizophrenia patients are labeled in red letters, and controls in blue. **b** Immunohistochemistry of NG2 in the frozen cortical sections of healthy controls and schizophrenia patients. Arrowheads and asterisks highlight NG2^+^ OPCs. **c** Quantification of NG2^+^ OPC number in the cortex. **d** Sholl analysis of NG2^+^ OPC in the cortex. **e** Immunohistochemistry of NG2 in the frozen hippocampal sections of healthy controls and schizophrenia patients. Arrowheads and asterisks highlight NG2^+^ OPCs. **f** Quantification of NG2^+^ OPC number in the hippocampus. **g** Sholl analysis of NG2^+^ OPC in the hippocampus. Plots show individual data and mean ± SD, or mean ± SEM in Sholl analysis results. n.s., not significant, ****p < 0.0001; two-sided Student’s t-test, or two-way ANOVA for Sholl analysis.


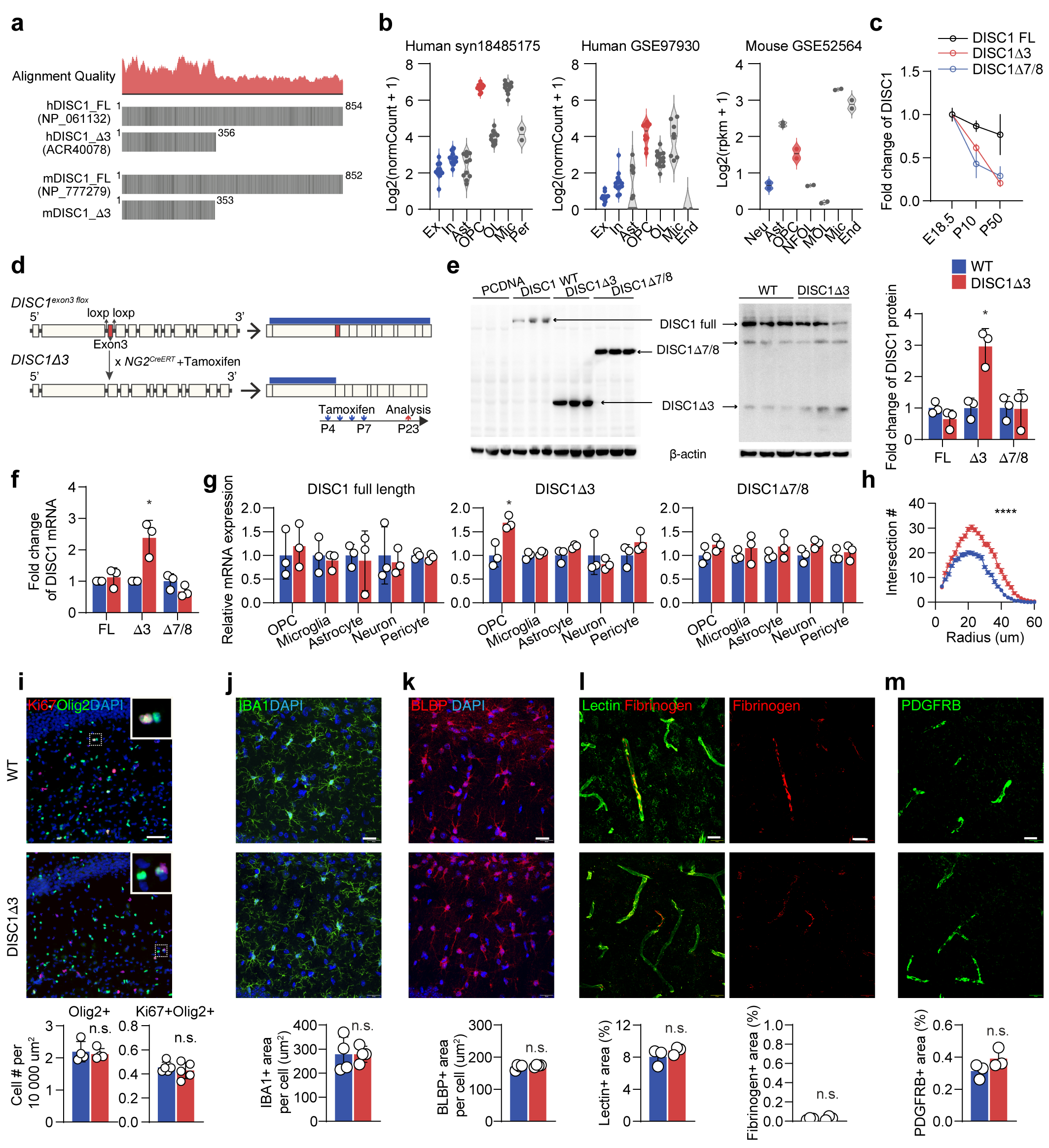


**Supplementary figure 2.** **The characteristics of DISC1 mRNA expression and abnormalities of other cell types in DISC1-Δ3 mice. a** Human and mouse DISC1 (full length and Δ3 variant) protein sequence alignment using ClusterW shows high homology. Human and mouse DISC1-Δ3 results in premature translation termination at the same site. Dark lines highlight mismatched amino acid residues. **b** Single-cell RNAseq analysis of DISC1 expression pattern in human and mouse. Ex, excitatory neuron; In, inhibitory neuron; Ast, astrocyte; OPC, oligodendrocyte precursor cell; OL, oligodendrocyte; Mic, microglia; Per, pericyte; End, endothelium; Neu, neuron; NFOL, newly-formed oligodendrocyte; MOL, mature oligodendrocyte. Blue color: neurons and red color: OPCs. **c** Expression of DISC1 splice variants at different stages in wildtype mice (E18.5, p10, p50). n = 3 mice from each timepoint. **d** DISC1 exon 3 deletion induced by tamoxifen (TAM) in NG2^CreERT^:Disc1^exon3 flox^ mice results in premature termination (blue lines highlight the coding region). Mice treated with TAM from P4 to P7 were subjected to analysis from P23. **e** Western blot of DISC1 splice variants expression in DISC1-Δ3 mouse brain tissues, showing that expression of DISC1-Δ3 was upregulated in our mouse model, while that of full length (FL) DISC1 or DISC1-Δ7/8 variant was unaltered. n = 3 mice. The left western blot of DISC1 in different DISC1 splice variants overexpressing 293T cells in order to verify the size of protein products of different variants in WT and DISC1-Δ3 mouse brain tissues. **f** RT-PCR of DISC1 splice variants expression in DISC1-Δ3 mouse brain tissues, showing that expression of DISC1-Δ3 was upregulated in our mouse model, while that of full length (FL) DISC1 or DISC1-Δ7/8 variant was unaltered. n = 3 mice. **g** RT-qPCR of DISC1 full length and splice variants expression in acutely isolated OPC, microglia, astrocyte, neuron and pericyte. n = 3 mice. **h** Sholl analysis of PDGFRα staining in WT and DISC1-Δ3 mice at P25. n = 30 cells from 4 mice. error bar: SEM. **i** Staining of Ki67/Olig2 in brain slice of DISC1-Δ3 mice at P25 and quantification of Olig2 in the hippocampus. Scale bar: 50 µm. n = 4 mice. Quantification of Ki67^+^ Olig2^+^ cell number in the hippocampus. n = 5 mice. **j** Staining of microglia (IBA1) and quantification of IBA1^+^ area in hippocampus. Scale bar, 20 µm. n = 4 mice. **k** Staining of astrocytes (BLBP) and quantification of BLBP^+^ area. Scale bar, 20 µm. n = 4 mice. **l** Staining of vasculature (Lectin and Fibrinogen), quantification of Lectin+ area, and quantification of Fibrinogen^+^ area outside of vessels. Scale bar, 20 µm. n = 3 mice. **m** Staining of pericytes (PDGFRβ) and quantification of PDGFRβ^+^ area. Scale bar, 20 µm. n = 3 mice. Plots show individual data and mean ± SD, n.s., not significant; *p < 0.05, ****p<0.0001; two-sided Student’s t-test, or two-way ANOVA for sholl analysis.


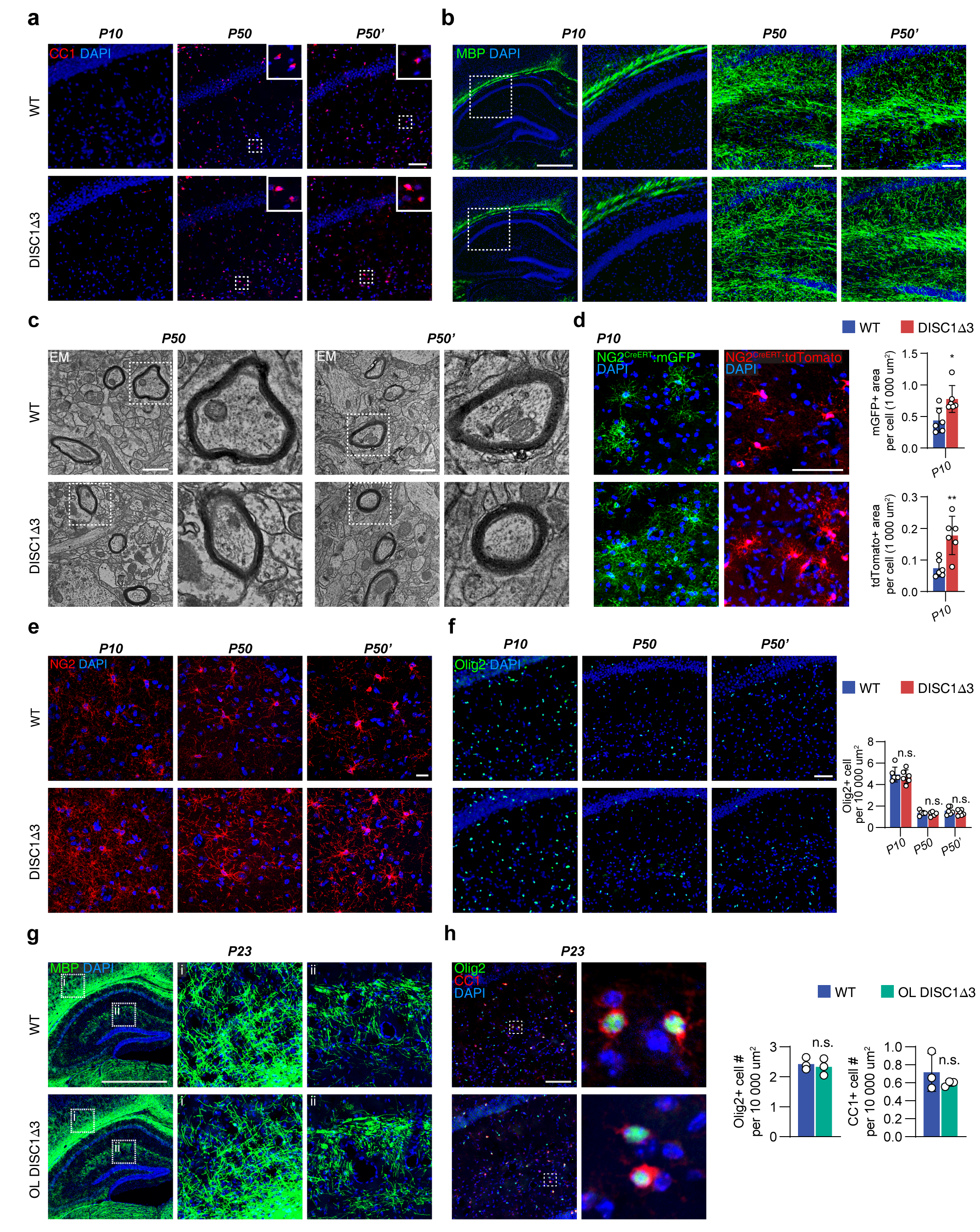


**Supplememtary figure 3. Neither oligodendrocyte maturation nor myelination was altered in different experimental settings. a** Staining of CC1 in DISC1-Δ3 mice in three different experiment setups. Scale bar, 50 µm. **b** Staining of MBP. Scale bar, 500 µm and 50 µm. **c** Electron microscopy image in hippocampus sections of the second and the third setups. Scale bar, 1 µm. **d** Imaging of hippocampal section of NG2^CreERT^:mT/mG or NG2^CreERT^:tdTomato mice in the first setups. Scale bar, 50 µm. Right panel, quantification of GFP^+^ or tdTomato^+^ area per cell in P10 NG2^CreERT^:mT/mG or NG2^CreERT^:tdTomato mice. n = 6 mice. **e** Immunostaining of NG2 in three different experiment setups. Scale bar, 10 µm. **f** Staining of Olig2 and quantification of Olig2^+^ cell number in the hippocampus. Scale bar, 50 µm. n = 5 or 6 mice. **g** Staining of MBP in OL DISC1-Δ3 mice. Region i and ii are enlarged at the right side, showing no myelin deficit in OL DISC1-Δ3 mice at P23. **h** Staining and quantification of Olig2 and CC1 in OL DISC1-Δ3 mice. Plots show individual data and mean ± SD, *p<0.05, **p<0.01, n.s., not significant; two-sided Student’s t-test.


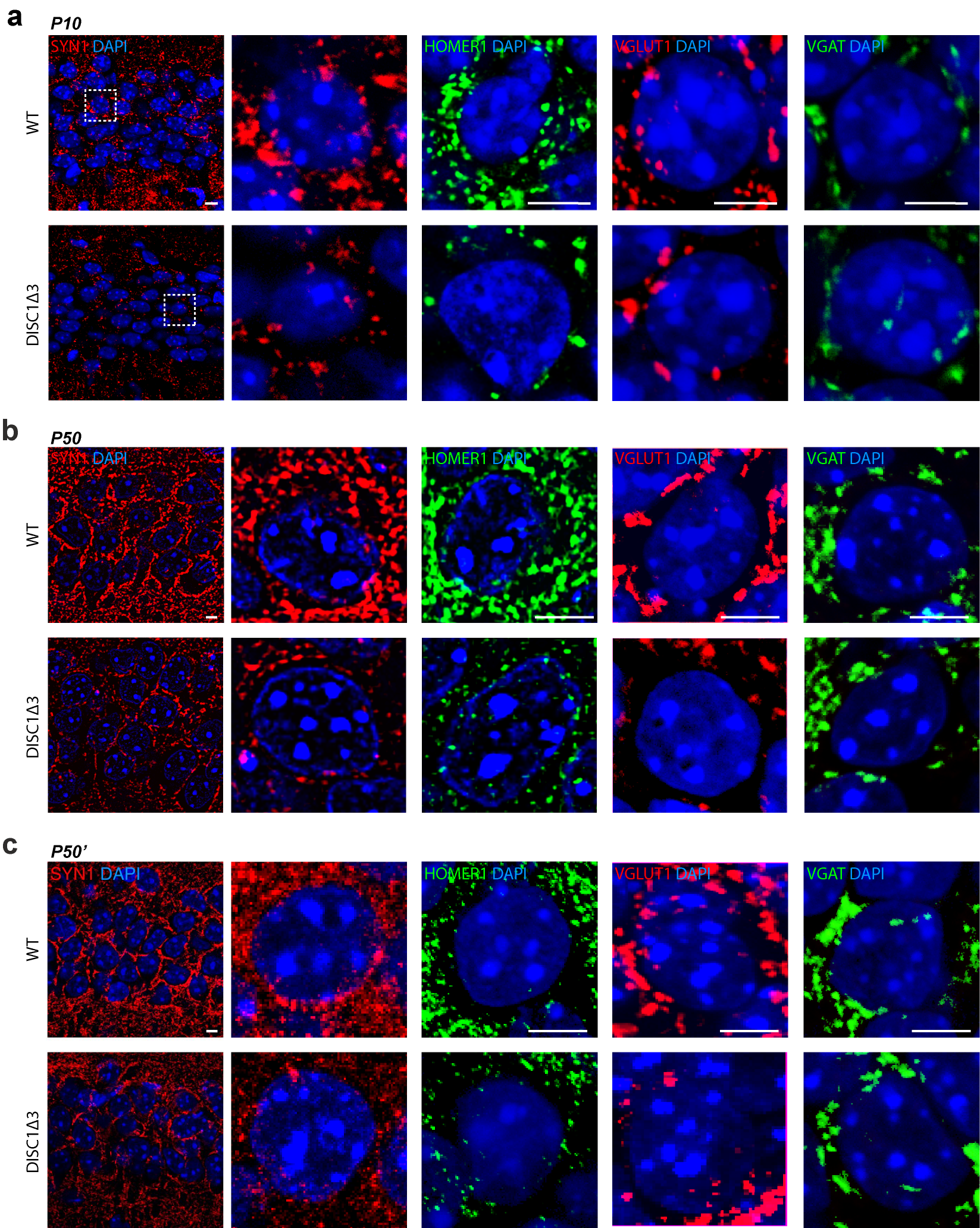


**Supplementary figure 4. Reduced synaptic markers in** **DISC1-Δ3 mice in the different experiment settings. a** Staining of SYN1, HOMER1, VGLUT1 and VGAT at P10. **b** Staining of SYN1, HOMER1, VGLUT1 and VGAT at P50. **c** Staining of SYN1, HOMER1, VGLUT1 and VGAT at P50 in DISC1-Δ3 mice with late tamoxifen induction (P40-P45). Scale bar, 5 µm.


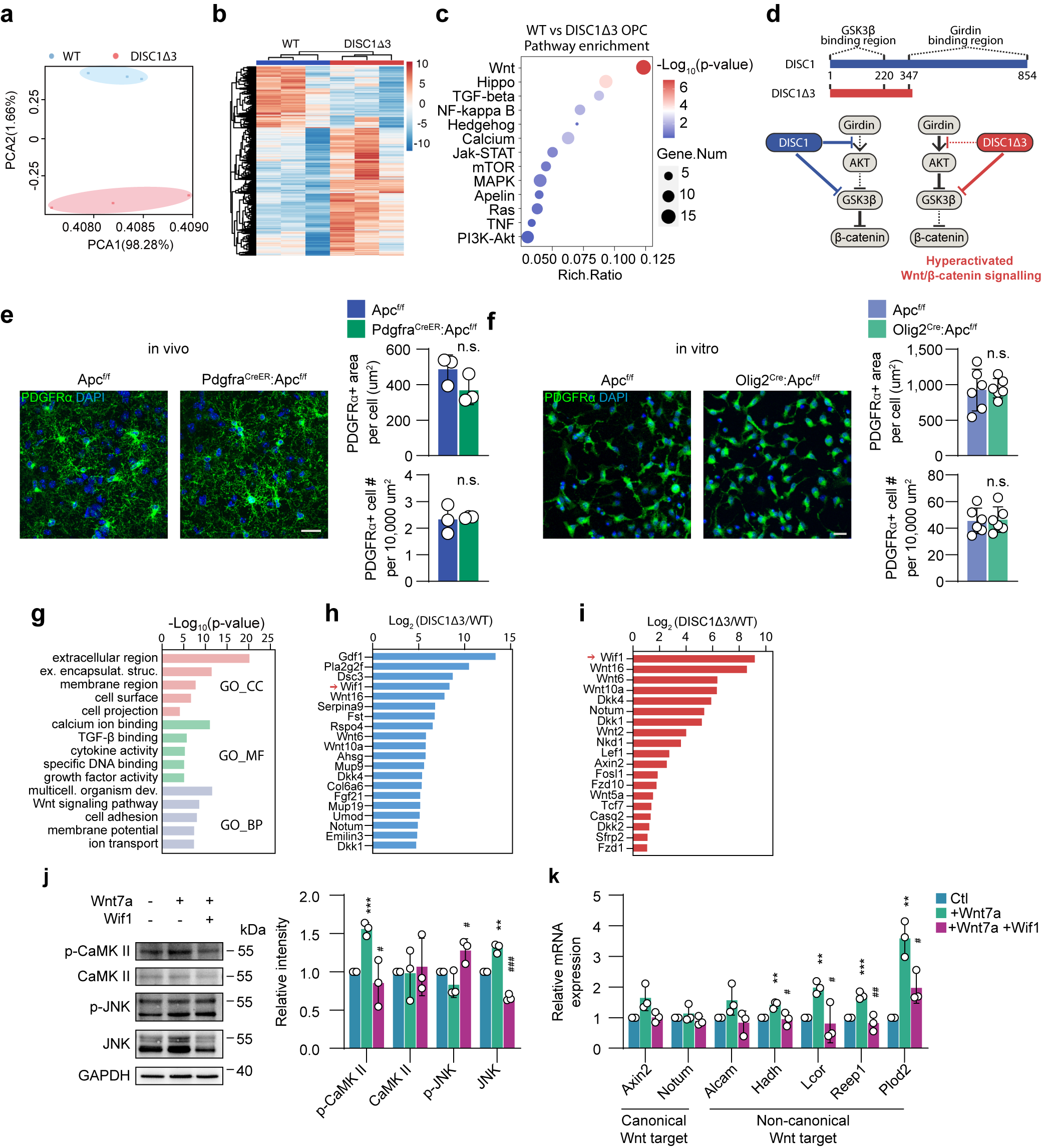


**Supplementary figure 5. Aberrant Wnt/β-catenin pathway activation in DISC1-Δ3 OPCs upregulates the expression of Wif1. a** PCA of differentially expressed genes in RNA-seq of WT and DISC1-Δ3 OPCs. **b** Heatmap of differentially expressed genes in RNA-seq of WT and DISC1-Δ3 OPCs. **c** KEGG pathway analysis of differentially expressed genes revealed by RNA-seq between acutely isolated WT and DISC1-Δ3 OPCs. **d** Model of DISC1-Δ3 regulation on Wnt/β-catenin signaling pathway. DISC1-Δ3 protein retains the GSK3β binding region but not the Girdin binding region. Both full length DISC1 and DISC1-Δ3 bind with GSK3β, directly inhibiting its activity, whereas DISC1-Δ3 could not bind to Girdin to inhibit AKT activity, leading to further suppression of GSK3β activity and subsequent hyperactivated Wnt/β-catenin signaling. **e** Staining and quantification of PDGFRα in control and Pdgfra^CreER^:Apc-floxed mice. Scale bar, 20 µm. n = 3 mice. **f** Staining and quantification of PDGFRα in primary OPC culture from control and Apc conditional knockout mice (n = 6 independent experiments). Scale bar, 20 µm. n = 6 experiments. **g** Gene ontology (GO) analysis of differentially expressed genes in RNA-seq of WT and DISC1-Δ3 OPCs. **h** The extracellular region-associated genes upregulated in DISC-Δ3 OPCs shown by RNA-seq. **i** Wnt pathway-related genes are upregulated in DISCΔ3 OPCs shown by RNA-seq. Arrow highlights Wif1. **j** Western blot and quantification of CaMK II and JNK phosphorylation in protein lysates from primary hippocampal neurons cultured with or without Wnt7a or Wif1. n = 3 experiments. **k** RT-qPCR on primary hippocampal neuronal culture to detect expression of canonical and non-canonical Wnt pathway target gene expression. n = 3 experiments. Plots show individual data and mean ± SD; n.s., not significant; *p < 0.05, **p < 0.01, ***p < 0.001; #, statistical analysis between Wnt7a treatment and Wnt7a+Wif1 treatment. #p < 0.05, ##p < 0.01, ###p < 0.001; two-sided Student’s t-test.


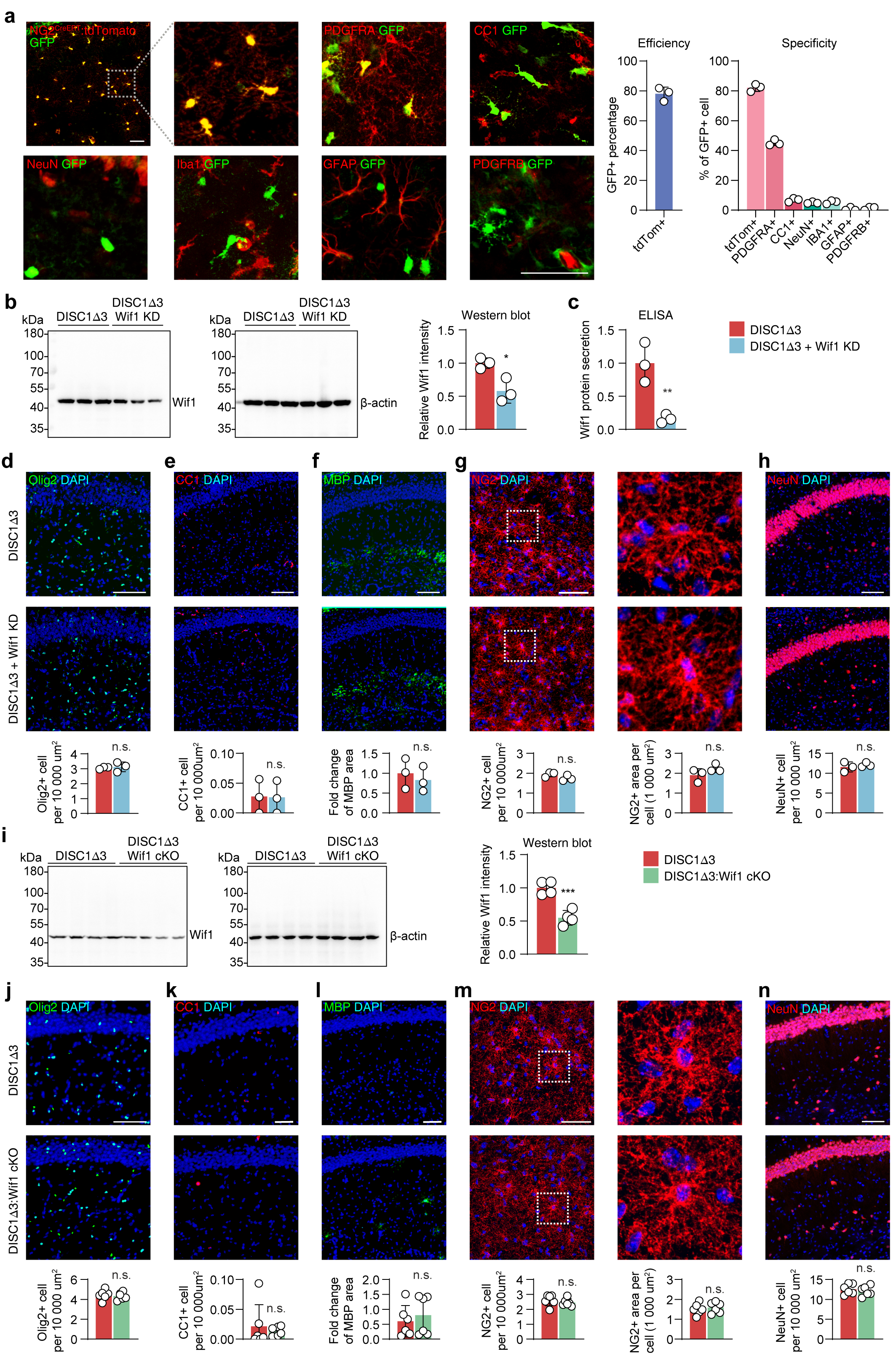


**Supplementary figure 6. Suppressing Wif1 expression in DISC-Δ3 OPCs affected neither oligodendrocyte maturation nor neuronal numbers. a** Staining and quantification of GFP-encoded OPC-preferential retrovirus efficiency and specificity. Cell type makers (OPC: NG2^creERT^:tdTomato, OL: CC1, microglia: IBA1, astrocytes: GFAP, neurons: NeuN, pericytes: PDGFRβ). GFP labels the transfected cells. Scale bar, 50 µm. n = 3 mice. **b** Western blot and quantification of Wif1 in the hippocampal lysate in the Wif1 KD experiments. n = 3 mice. **c** Detection of Wif1 protein expression in the hippocampus using ELISA. n = 3 mice. **d-h** Immunostaining and quantification of Olig2 (d), CC1 (e), MBP (f), NG2 (g), NeuN (h) in CA1 region of DISC-Δ3 mice with or without Wif1 KD. Scale bar, 50 µm. n = 3 mice. **i** Western blot and quantification of Wif1 in the hippocampal lysate in the Wif1 conditional KO experiments. n = 4 mice. **j-n** Immunostaining and quantification of Olig2 (j), CC1 (k), MBP (l), NG2 (m), NeuN (n) in CA1 region. Scale bar, 50 µm. n = 6 mice. Plots show individual data and mean ± SD; n.s., not significant, * p<0.05, ** p<0.01, *** p<0.001; two-sided Student’s t-test.
